# Precision mRNA Delivery via Ultrasound‐Controlled Release Perfluorocarbon Emulsions: An Innovative Ultrasound Theranostic Strategy with ^19^F MRI Feasibility

**DOI:** 10.1002/smll.202506806

**Published:** 2025-11-28

**Authors:** Haikun Liu, Mark Louis P. Vidallon, Yuyang Song, Aidan P. G. Walsh, Henry Gordon, Shulei Ren, Pengkai Shi, Bangyan Xu, Mitchell J. Moon, Sylvain Trépout, Rico F. Tabor, Alexis I. Bishop, Ulrich Flögel, Karlheinz Peter, Xiaowei Wang

**Affiliations:** ^1^ Molecular Imaging and NanoTherapeutics Laboratory Baker Heart and Diabetes Institute 75 Commercial Road Melbourne VIC 3004 Australia; ^2^ Centre for Cardiometabolic mRNA Therapy Baker Heart and Diabetes Institute 75 Commercial Road Melbourne VIC 3004 Australia; ^3^ Department of Cardiometabolic Health University of Melbourne Parkville VIC 3010 Australia; ^4^ School of Chemistry Monash University Clayton VIC 3800 Australia; ^5^ Baker Department of Cardiovascular Research Translation and Implementation La Trobe University Bundoora VIC 3086 Australia; ^6^ Atherothrombosis and Vascular Biology Laboratory Baker Heart and Diabetes Institute 75 Commercial Road Melbourne VIC 3004 Australia; ^7^ Ramaciotti Centre for Cryo‐electron Microscopy Monash University Clayton VIC 3800 Australia; ^8^ School of Physics and Astronomy Monash University Clayton VIC 3800 Australia; ^9^ Institute for Molecular Cardiology Medical Faculty and University Hospital Düsseldorf Heinrich‐Heine‐University Düsseldorf Universitätsstraße 1 40225 Düsseldorf Germany; ^10^ Cardiovascular Research Institute Düsseldorf (CARID) Medical Faculty and University Hospital Düsseldorf Heinrich‐Heine University Düsseldorf Universitätsstraße 1 40225 Düsseldorf Germany; ^11^ School of Translational Medicine Monash University Melbourne VIC 3004 Australia

**Keywords:** magnetic resonance imaging, mRNA, perfluorocarbon nanodroplets, theranostic, ultrasound

## Abstract

Messenger RNA (mRNA) therapeutics provide promising opportunities in cardiovascular diseases. However, effective vascular mRNA delivery requires precise delivery, controlled release, and efficient transfection. To address these challenges, the study utilizes phase‐change perfluorocarbon nanodroplets (PFC NDs) as a unique theranostic platform, integrating multimodal imaging with ultrasound‐triggered mRNA release for enhanced vascular transfection. Lipid‐coated PFC NDs are engineered using perfluoro‐crown‐ether (PFCE), perfluorohexane (PFH), and perfluoropentane (PFP) as core materials. These nanodroplets (200–300 nm) are optimized for mRNA loading and cellular uptake, exhibiting strong ultrasound contrast in tissue‐mimicking phantoms and in vivo. Each PFC generated distinct fluorine‐19 magnetic resonance imaging signals, allowing tri‐spectral imaging capabilities. In vitro, PFH and PFP NDs increased enhanced green fluorescent protein mRNA transfection in CHO cells (*p* < 0.0001), with ultrasound stimulation further improving efficiency compared to unstimulated controls (*p* < 0.05). In vivo ultrasound‐guided activation of PFH NDs resulted in higher mCherry protein expression in murine carotid arteries (*p* < 0.05), demonstrating site‐specific gene therapy. This study establishes PFH NDs as an advanced theranostic platform for vascular mRNA delivery, integrating diagnostics and therapeutics into a single system. By leveraging ultrasound‐responsive activation, these nanodroplets overcome existing delivery limitations, offering a new avenue for precision medicine in cardiovascular disease.

## Graphical abstract

A key challenge in advancing messenger RNA (mRNA) therapeutics in cardiovascular diseases lies in achieving controlled release at the precise site within the vasculature. This study presents the development of perfluorocarbon nanodroplets with exceptional theranostic potential, enabling real‐time tracking through ultrasound and fluorine‐19 (^19^F) magnetic resonance imaging. Moreover, ultrasound‐triggered activation enhances mRNA delivery, making these colloids promising candidates for image‐guided cardiovascular therapies.

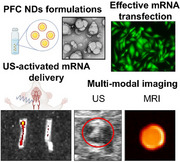

## 1 Introduction

Advancements in nanomaterials and messenger RNA (mRNA) technologies have the potential to enable transformative breakthroughs ranging from mRNA‐based vaccines to novel therapeutics applications. mRNA‐based therapeutics are remarkably versatile, offering approaches such as immunotherapies, protein‐replacement therapies, chimeric antigen receptor T‐cell (CAR T) therapies, and CRISPR‐associated therapies to address diseases ranging from infections and cancers to genetic and cardiovascular conditions.^[^ ^1^ ^]^ However, despite their potential, mRNA therapeutics face distinct challenges compared to vaccines. Unlike vaccines, which require minimal protein expression to trigger an immune response, mRNA therapies may demand up to 1000 times higher protein levels for efficacy. Additionally, while mRNA vaccines rely on simple intramuscular injections, specific therapeutic mRNAs require specific organs, tissues, or cellular delivery.^[^ ^2^ ^]^

In the context of cardiovascular diseases (CVDs), mRNA therapeutics have shown promise in addressing conditions such as hypercholesterolemia, atherosclerosis, and heart failure. Vascular endothelial growth factor (VEGF) mRNA has been considered one of the promising candidates for heart failure treatment.^[^ ^3^ ^]^ In a phase II clinical trial (NCT03370887), the intramyocardial injection of naked VEGF mRNA (AZD8601) demonstrated safety without adverse events in patients undergoing coronary artery bypass surgery. However, the trial was not powered to assess therapeutic efficacy.^[^ ^4^ ^]^ Several mRNA and/or gene therapeutic targets for combating atherosclerosis have also been identified, such as BCL‐2‐associated X protein,^[^ ^5^ ^]^ interleukin‐10,^[^ ^6^ ^]^ and Krüppel‐like factor 2.^[^ ^7^ ^]^ However, these strategies hinge on developing effective targeting and delivery technologies to the vascular walls, areas that remain underexplored and require further innovation to fully realize the potential of mRNA therapeutics.

Currently, the delivery of mRNA is typically achieved with lipid nanoparticles (LNPs), mainly consisting of ionizable lipids. LNPs protect mRNA from degradation by nucleases, phagocytosis of immune cells and renal clearance, and help mRNA breach multiple membrane barriers to promote cell internalization and endosomal escape.^[^ ^8^ ^]^ Recent advancements for mRNA‐targeted delivery via LNPs primarily rely on two methods. The first involves adjusting lipid components to alter the organ tropism of LNPs, as seen using selective organ targeting (SORT) nanoparticles.^[^ ^9^ ^]^ However, LNP formulations specifically targeting atherosclerotic plaque have not yet been developed. The second strategy employs functionalization with antibodies or peptides. For example, micro/nanoparticles targeting VCAM‐1 or activated platelets have shown successful binding to plaques^[^ ^10^ ^]^ and thrombi,^[^ ^11^, ^12^ ^]^ respectively. While functionalization is effective, it requires specific antibody or peptide design, and sometimes multiple conjugation steps (reaction and purification), adding several layers of fabrication process complexity.

External stimuli‐enhanced drug delivery presents an attractive alternative for vascular targeting, in particular, ultrasonic stimulation.^[^ ^13^, ^14^, ^15^ ^]^ Among clinical approaches, ultrasound has gained significant attention due to its widespread clinical availability, cost‐effectiveness, non‐invasiveness, real‐time imaging capability, and high safety profile.^[^ ^14^ ^]^ Both microbubbles and nanodroplets can be used as ultrasound‐responsive drug delivery systems.^[^ ^13^, ^14^, ^16^, ^17^ ^]^ Under ultrasound stimulation, microbubbles can facilitate mRNA transfection via stable cavitation (stable bubble oscillations) or inertial cavitation (violent bubble implosion and collapse). Microbubble oscillations exert mechanical forces on biological barriers, such as cell membranes, thereby increasing cell permeability, while microbubble collapse can induce additional mechanical effects, such as shockwaves and microjets, further improving delivery.^[^ ^14^, ^16^ ^]^ However, the low stability of current clinically used microbubbles and limited circulation in the body may hinder their therapeutic application.

Perfluorocarbon (PFC) nanodroplets (NDs) have emerged as an alternative for microbubbles. These ultrasmall liquid droplet dispersions contain a phase‐change PFC core and typically a surfactant shell, functioning as both delivery vehicles and microbubble precursors. Under ultrasound stimulation, PFC undergoes liquid‐to‐gas phase transition, converting NDs to microbubbles through acoustic droplet vaporization (ADV), allowing them to be visualized on ultrasound diagnostic imaging.^[^ ^18^, ^19^, ^20^ ^]^ Compared with microbubbles, NDs exhibit superior stability and distribution in biological systems due to their smaller size and dense liquid core,^[^ ^16^, ^19^ ^]^ enhancing their potential for mRNA delivery, particularly in vascular applications.

Beyond drug delivery and visualization by ultrasound, PFC NDs are employed in preclinical works as contrast agents for fluorine‐19 magnetic resonance imaging (^19^F MRI),^[^ ^21^, ^22^ ^]^ positioning them as theranostic particles for simultaneous diagnostic imaging and site‐specific therapy. The fluorine content of PFCs allows highly specific, background‐free contrast in ^19^F MRI, and when combined with ^1^H MRI, it provides the precise anatomical localization of PFC NDs.^[^ ^22^ ^]^

PFC NDs have been explored as potential ultrasound‐controlled drug delivery systems for cancers and inflammatory diseases.^[^ ^23^, ^24^, ^25^, ^26^, ^27^ ^]^ However, their applications for vascular wall‐targeting CVD treatment remain underexplored, and their potential as mRNA delivery systems to the cardiovascular system has never been reported yet. In this study, we developed three distinct PFC NDs based on classical mRNA‐delivery liposome formulation containing 1,2‐dioleoyl‐sn‐glycero‐3‐phosphoethanolamine (DOPE) and cationic 3β‐[*N*‐(*N*“,*N*”‐dimethylaminoethane)‐carbamoyl] cholesterol (DC‐cholesterol) as the lipid shell, and incorporating different PFC cores: perfluoro‐crown‐ether (PFCE), perfluorohexane (PFH), and perfluoropentane (PFP). With varying alkyl chain lengths^[^ ^28^ ^]^ and bulk boiling points (T_b_ of PFCE, PFH, and PFP are 145, 56, and 29 °C, respectively), we demonstrated their difference in stabilities during ultrasound imaging and burst ultrasound‐responsiveness for on‐demand mRNA transfection. By evaluating their imaging properties and mRNA transfection capabilities both in vitro and in vivo, our work highlights the versatility and adaptability of PFC NDs, demonstrating their highly promising potential for innovative mRNA delivery technologies and theranostic approaches for site‐specific vascular targeting.

## 2 Results and Discussion

### 2.1 Fabrication, Optimization, and Structural Characterization of PFC NDs

**Figure 1:**
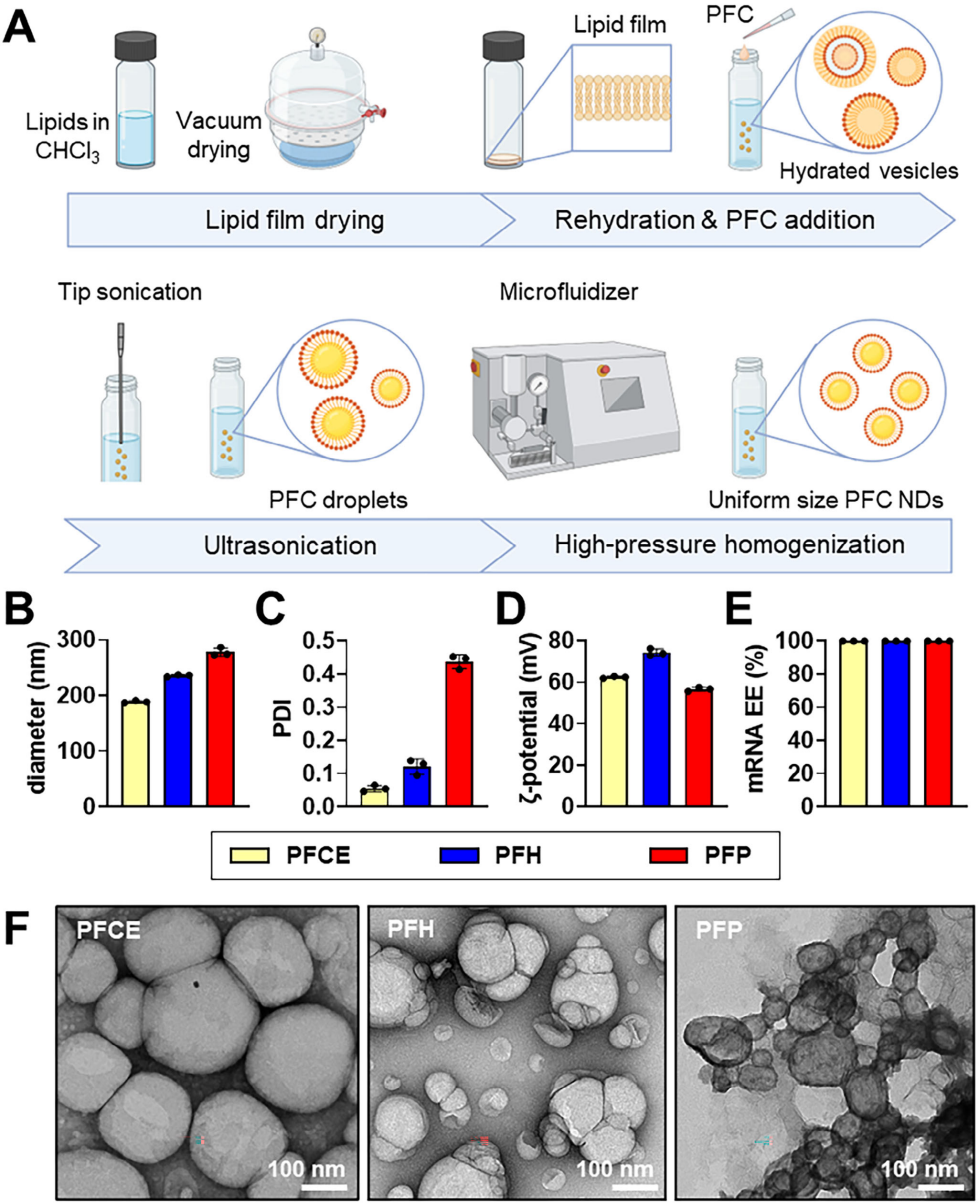
Fabrication and characterization of PFC NDs. A) Schematic diagram of PFC ND fabrication process (Created with BioRender.com). B) Size, C) PDI, and D) zeta potential of the PFCE, PFH, and PFP NDs. E) mRNA entrapment efficiency (EE) data from Ribogreen assay. F) Transmission electron micrographs of negatively stained PFCE, PFH, and PFP NDs. Scale bars = 100 nm.

PFC NDs were fabricated using a modified thin film–hydration–sonication method, followed by high‐pressure homogenization using a microfluidizer (**Figure** **1**A). Different lipid shell compositions were tested in order to achieve properties based on a specific set of design criteria, ideal for ultrasound‐guided mRNA delivery and ultrasound‐mediated targeted delivery. mRNA transfection and material contrast in ultrasound imaging are generally affected by the droplet size and acoustic impedance mismatch with the target biological system (for contrast‐enhanced ultrasound imaging).^[^ ^29^ ^]^ These properties were measured using dynamic light scattering (DLS, for droplet size), electrophoretic light scattering (ELS, for zeta potential), and brightness (B)‐mode ultrasound imaging (for ultrasound contrast enhancement).

In our design criteria, an ideal droplet size range for ND‐mediated mRNA therapies is 200–300 nm. This range is rooted in theoretical considerations aimed at ensuring effective cellular uptake, consequently leading to high transfection efficiency and responsiveness to ultrasound stimuli. Generally, smaller nanomaterials possess enhanced capability to traverse cell membranes through passive uptake. Nonetheless, it is crucial to note that smaller nanoparticles also exhibit heightened cytotoxicity compared to their larger counterparts.^[^ ^30^ ^]^ Previous studies have demonstrated that nanomaterials sized between 200–300 nm can still be efficiently internalized into cells via caveolae‐mediated pinocytosis.^[^ ^31^ ^]^ This size range strikes a delicate balance between functionality and responsiveness. Smaller droplet sizes notably elevate PFCs’ resonance frequency^[^ ^32^ ^]^ and boiling point (via increased Laplace pressure),^[^ ^33^, ^34^, ^35^ ^]^ subsequently demanding higher energy input to induce acoustic droplet vaporization.^[^ ^36^ ^]^ Moreover, smaller droplets exhibit weaker acoustic backscatters compared to larger ones, rendering extremely diminutive materials unsuitable as ultrasound contrast agents. In terms of zeta potential, it is desirable to have highly positive values, ideally above +20 mV, which both indicate good colloidal stability and strong positive surface charge that is important for mRNA capture and surface immobilization. Based on the results of the optimization (Table S1, Supporting Information), the formulation with 2:1 DOPE: DC‐cholesterol and 5% PFCE was found to have droplet diameters within the target size range, in addition to the strong ultrasound contrast in B‐mode imaging with the longest contrast peak times. Furthermore, this PFC ND formulation exhibited a strong positive surface charge. Hence, we chose the DOPE–DC‐cholesterol system (2:1 mass ratio) as the optimal lipid shell system.

Following lipid shell optimization using PFCE as the core, we created a set of DOPE–DC‐cholesterol‐shelled PFC NDs using PFCE, PFH, and PFP. These PFCs are commercially available and have been used previously in a variety of biomedical applications, including oxygen carriers,^[^ ^37^ ^]^ contrast agents for ultrasonography and MRI.^[^ ^38^ ^]^ Based on DLS, the average diameters of PFCE NDs, PFH NDs, and PFP NDs were 188.8 ± 2.0, 235.9 ± 2.0, and 277.9 ± 7.4 nm, respectively (Figure 1B), which are ideal size range for ND‐mediated mRNA therapies. PFCE and PFH NDs have relatively low polydispersity indexes (PDI), indicating reasonable size homogeneity, as compared to PFP NDs with high PDI value of ≈0.4 (Figure 1C). This broader distribution reflects the intrinsic volatility and low boiling point (29 °C) of PFP, which makes it prone to partial vaporization, coalescence, and deformation during emulsification and handling, thereby reducing size uniformity. In contrast, PFCE (145 °C) and PFH (56 °C) possess higher thermal stability, supporting more consistent droplet formation.

Zeta potential analysis showed positive surface charge of +62.4 ± 0.5, +74.3 ± 1.7, and +56.6 ± 1.0 mV for PFCE NDs, PFH NDs, and PFP NDs, respectively (Figure 1D). The positive surface charge of the PFC NDs facilitates electrostatic complexation with polyanionic mRNA. Quantification by a modified RiboGreen assay (Figure 1E) revealed an entrapment efficiency of 99.9% across all PFC NDs, indicating complete mRNA loading at the experimental ratio used. Notably, variations in PFC core composition and size did not affect mRNA complexation capacity.

TEM images of negatively stained PFC NDs in Figure 1F corroborate the DLS results. All ND formulations had generally spherical morphologies with some structural variations, depending on PFC content. Notably, PFCE NDs exhibit the most uniform size and morphology, whereas PFH and particularly PFP NDs showed greater structural variability (Figure S1, Supporting Information), including size distribution, coalescence, and buckling or collapse. The low boiling point and high volatility of PFP make it more susceptible to deformation, partial evaporation, and collapse during both emulsification and TEM sample preparation under vacuum. This behavior reflects instability under imaging conditions rather than true disintegration of droplets in suspension, highlighting the critical influence of PFC core volatility on emulsion uniformity and imaging artefacts.

Storage stability studies over 30 days at 4 °C (Figure S2, Supporting Information) provided additional insights into the physicochemical stability of PFC NDs. No significant differences in size or zeta potential were observed over the 30 days. Collectively, these results highlight the influence of PFC core properties on ND uniformity and stability, which is critical for their subsequent evaluation in ultrasound‐triggered mRNA delivery and imaging applications.

### 2.2 Bio‐ and Hemocompatibility of PFC NDs

Biological safety of PFC NDs was evaluated using two in vitro approaches: 1) evaluation of viability of model cells after acute exposure to PFC NDs via an MTT assay; and 2) assessment of compatibility with blood and blood cells. MTT assays against Chinese hamster ovarian (CHO) cells were performed to evaluate cytocompatibility of PFC NDs, particularly their effect on the metabolic capabilities of the model cells.

**Figure 2:**
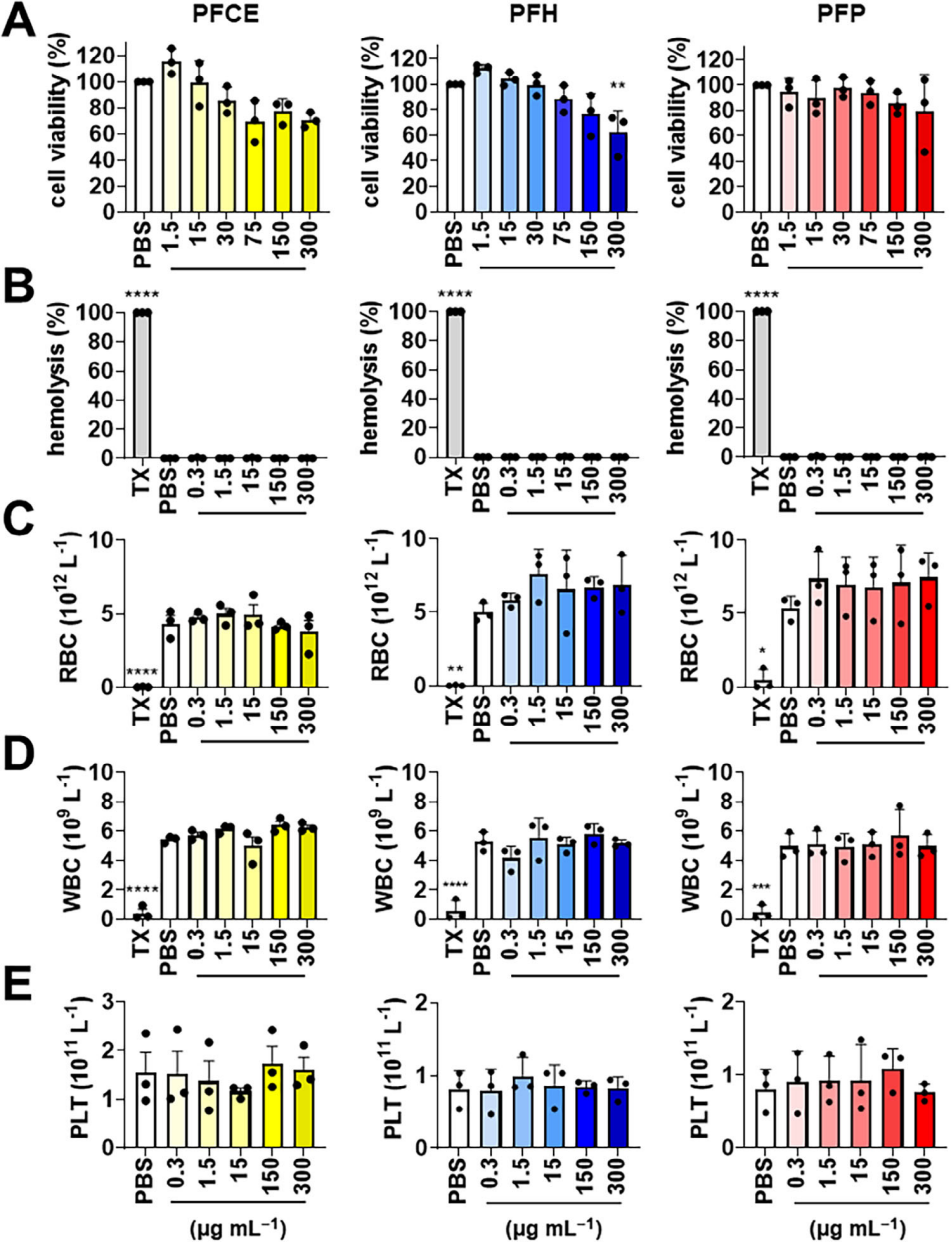
Bio/hemocompatibility of PFCE, PFH, and PFP NDs: A) MTT cell viability of PFC ND‐treated CHO cells; B) hemolysis, C) RBC, D) WBC, and E) platelet counts of blood samples treated with PFC NDs. Triton X‐100 (TX) as hemolysis positive control and PBS as vehicle control (untreated). Data presented as mean ± SD (*n* = 3 independent experiments with 3 blood donors for hemocompatibility experiments); *p < 0.05, **p < 0.01, ***p < 0.001, ****p < 0.0001 by Brown–Forsythe and Welch ANOVA with Dunnett's multiple comparisons test, comparing all groups. The P values presented indicate comparison against the PBS control.

As indicated in **Figure** **2**A, all three PFC NDs showed a dose‐dependent reduction in CHO cell viability. All PFC ND formulations showed a wide range of concentrations with no significant cell viability reduction. It is important to highlight that the test concentration (maximum 15 µg mL^−1^) for all transfection experiments conducted in this work falls within the safe and cytocompatible range for CHO cells. This broad safety window underscores the versatility of the PFC ND formulations, allowing their use across various in vitro applications, as well as potential biomedical applications, requiring different dosage levels. It is also important to emphasize that higher concentrations tested, especially for PFH NDs (300 µg mL^−1^), where cell viability decrease was observed, are deliberately set at elevated, suprapharmacological doses, 10 to 20 times the concentrations used for transfection experiments. These doses are chosen to illustrate the limits of the model and represent extreme conditions intentionally employed for demonstration purposes.

Evaluating hemocompatibility is crucial in assessing the immediate interaction between blood and its components upon contact with PFC NDs, as these materials are specifically designed for intravenous administration in cardiovascular applications. Based on the result (Figure 2B–E), all three PFC NDs exhibited strong safety profiles; they did not lyse red blood cells (RBCs) nor influence the number of white blood cells (WBCs) and platelets after incubation with whole blood for 1 h at 37 °C. This outcome provides additional confirmation of the biocompatibility of PFC NDs, supporting their suitability for further drug delivery applications. Taking all of these into account, overall, these results indicate that PFC NDs can be administered at safe dosages that will not interfere with normal blood conditions and cell metabolism.

### 2.3 Imaging Properties of PFC NDs

The potential of PFC NDs as contrast agents for multimodal imaging via ultrasound (both in vitro and in vivo) and ^19^F MRI (in vitro), as well as their acoustic properties as colloidal agents for ultrasound‐activated mRNA theranostics, were investigated.

#### 2.3.1 In Vitro Acoustic Contrast Enhancement

The PFC ND formulations were placed in tissue‐mimicking phantom (2% agarose gel) to replicate the acoustic properties of soft tissues,^[^ ^33^, ^39^ ^]^ serving as a quick and efficient model for testing acoustically active colloidal materials.

**Figure 3:**
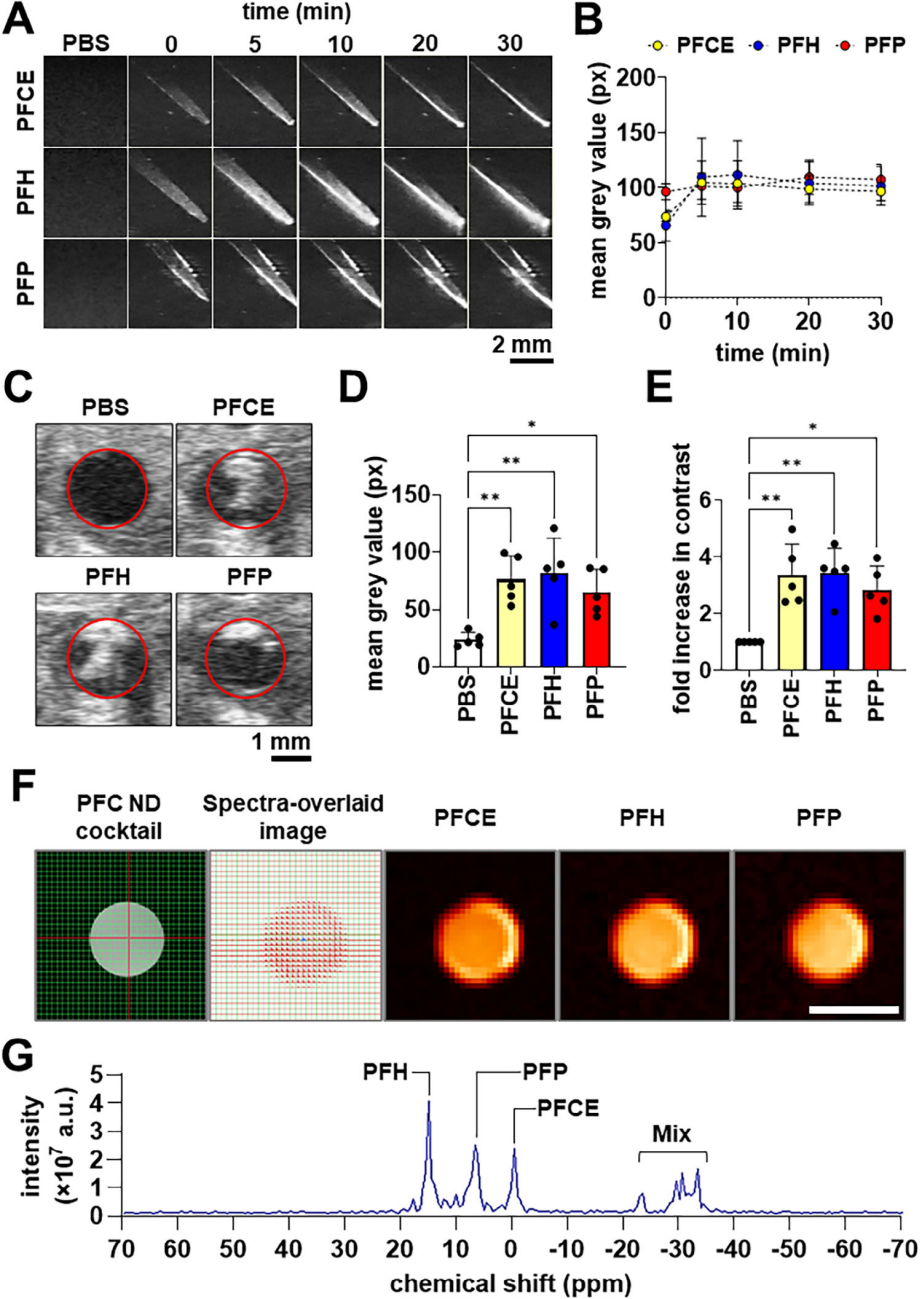
Ultrasound and ^19^F MR spectral unmixing of PFC NDs. A) B‐mode ultrasonograms of PFCE, PFH, and PFP ND‐loaded tissue‐mimicking phantoms showing the contrast evolution over a 30‐minute observation period. Scale bars = 2 mm. B) Mean grey values from ultrasonograms indicating ultrasound contrast enhancement of PFCE, PFH, and PFP NDs in tissue‐mimicking phantoms over a 30‐minute observation period. C) Representative B‐mode ultrasonograms showing the inferior vena cava of subject mice injected with PBS, PFCE NDs, PFH NDs, and PFP NDs. Red circles indicate the walls of the inferior vena cava. Scale bar = 1 mm. Bar graphs showing the ultrasound contrast enhancement by PFC NDs within the inferior vena cava, represented by D) mean grey value and E) fold increase in ultrasound contrast, relative to PBS. Bar graphs are shown as mean ± SD from three independent experiments (*n* = 3); *p < 0.05, **p < 0.01 by one‐way ANOVA with Tukey's multiple comparisons between all groups. F) ^19^F MR images of PFC ND mixture: axial cross‐section of the scan area (sample‐loaded tube) in PFC ND cocktail, with each image pixel representing individual MR spectra in the spectra‐overlaid image and reconstructed ^19^F images of PFCE, PFH, and PFP. Scale bar = 10 mm. G) Representative ^19^F MR spectral unmixing of the PFC NDs cocktail (PFH, PFP, and PFCE NDs) and labeled individual chemical shifts that separate their distinct PFC compound signatures.

**Figure** **3**A shows the B‐mode ultrasonograms of the tissue‐mimicking phantoms with and without PFC NDs (3 mg mL^−1^ PFCE, PFH, and PFP NDs) over a 30‐minute observation period. Visual contrast enhancement is evident in all PFC ND‐containing phantoms, possibly attributed to the acoustic impedance mismatch between the PFC cores and the phantoms (≈98% water). Note that acoustic impedance (*Z*) is the product of the density (ρ) of the material and the speed of sound (ν) through this material (mathematically expressed as *Z*  =  ρν). PFCs exhibit significantly greater densities than water (0.99 g cm^−3^): ρ (PFCE) = 1.78 g cm^−3^; ρ (PFH) = 1.76 g cm^−3^; and ρ (PFP) = 1.62 g cm^−3^.^[^ ^38^ ^]^ The increased intensity of the grey pixels at the bottom of the phantoms indicates the gravitational sedimentation of the PFC ND over time, confirming their higher density compared to water. Notably, PFP NDs exhibited reverberation artifacts—grey pixels outside the sample‐filled area resembling those inside—suggesting some bubble formation. Quantifying the mean grey value of the sample‐filled regions showed that all PFC ND formulations had similar, stable contrast intensification throughout the 30‐minute observation period (Figure 3B).

#### 2.3.2 In Vivo Ultrasound Imaging of PFC NDs

The ultrasound contrast enhancement provided by PFC NDs was further investigated in vivo using the inferior vena cava of live mice, following femoral vein injection. Figure 3C–E shows that the injections of PFC NDs (3 mg mL^−1^) significantly enhanced ultrasound contrast within the lumen of the inferior vena cava, with an average of 2.8‐ to 3.4‐fold enhancement observed. One notable observation in the ultrasonograms is the localized contrast enhancement around the upper part of the vessel lumen when mice were injected with PFP NDs, while PFCE and PFH NDs resulted in evenly distributed contrast within the lumen. These observations can be attributed to the differences in the stability of the PFC cores. While size reduction and confinement of PFCs within small NDs enhance their stability through Laplace pressure elevation,^[^ ^40^ ^]^ PFP (boiling point, T_b_ = 29 °C) undergoes partial heat‐induced phase transition into microbubbles due to the body temperature of mice exceeding 29 °C,^[^ ^41^ ^]^ which may contribute to shadowing on ultrasound imaging. This stability is reflected in heating experiments with optical microscopy and the thermogravimetric analysis results in the Supporting Information (Figure S3, Supporting Information). In contrast, PFH and PFCE NDs with higher bulk phase transition temperatures (T_b_ = 56 and 146 °C, respectively), maintained their structure as liquid NDs, ensuring greater dispersible and colloidally stable.

#### 2.3.3 In Vitro MRI Contrast Enhancement

The distinct chemical shift (δ) of the PFC cores enables multi‐color ^19^F MRI, facilitating the selective detection of individual PFC compounds. By employing targeted PFC emulsions to different biomarkers, we have previously demonstrated the ability to determine disease states by differentiating between the biological progress of inflammation, thrombosis, and fibrosis in a spontaneous myocardial infarction murine model.^[^ ^21^ ^]^ In this study, ^19^F MRI was performed using a cocktail of the three PFC NDs. Figure 3F demonstrates that each PFC ND produced distinct MR contrast signals, which were isolated based on their unique chemical shifts, enabling multi‐chemical selective imaging. As demonstrated in Figure 3G, the chemical shifts of PFCE, PFH, and PFP NDs corresponded to δ = −0.8, 7.5, and 17.0 ppm, respectively. This tricolor MRI capability allowed clear and simultaneous visualization of each PFC construct. Among the three, PFH NDs exhibited the highest signal intensity (146.1), followed by PFCE NDs (96.1) and PFP NDs (89.9). The PFC dose used in this work (≈2.3 × 10^−5^ mol per mouse) is comparable to that reported in previous small‐animal ^19^F MRI studies^[^ ^42^ ^]^ and remains well within the biocompatible range established in our earlier cardiovascular imaging work,^[^ ^21^ ^]^ where doses up to ≈6.0 × 10^−5^ mol per mouse were safely administered without observable toxicity.

While these results confirm the fluorine detectability of the PFC NDs, the current system primarily functions as an ultrasound‐based theranostic platform, where the nanodroplets can be visualized in real time and externally triggered to release their mRNA payload under ultrasound exposure. Furthermore, given the limited sensitivity of current clinical ^19^F MRI, simultaneous imaging and therapeutic delivery at a single, clinically acceptable dose is not yet feasible in humans. Therefore, the inclusion of ^19^F MRI in this study serves as complementary feature to demonstrate the intrinsic detectability of the fluorinated nanodroplets rather than simultaneous therapeutic operation.^[^ ^21^, ^22^ ^]^ Nevertheless, as advances in MRI probe sensitivity, pulse sequence design, and fluorine payload optimization emerge, this platform could evolve toward dual‐modality applications that combine ultrasound precision with quantitative MRI tracking, extending its translational potential.

### 2.4 mRNA Transfection with PFC NDs

#### 2.4.1 Direct Transfection

The efficiency of mRNA transfection using PFC NDs was evaluated in vitro using CHO cells. The model mRNA encoding enhanced green fluorescent protein (eGFP mRNA), was mixed and incubated with the PFC ND dispersions, allowing the mRNA to complex with the droplet interface through ion–dipole and electrostatic interactions, where the positively charged amino groups of DC‐cholesterol (with a pKa = 7.8)^[^ ^43^ ^]^ interacted with the negatively charged phosphate groups of the mRNA molecules. The “direct transfection” method involved the incubation of the eGFP mRNA‐loaded PFC NDs with the cells for 24 h at 37 °C. This approach provided insights into transfection efficiency.

**Figure 4:**
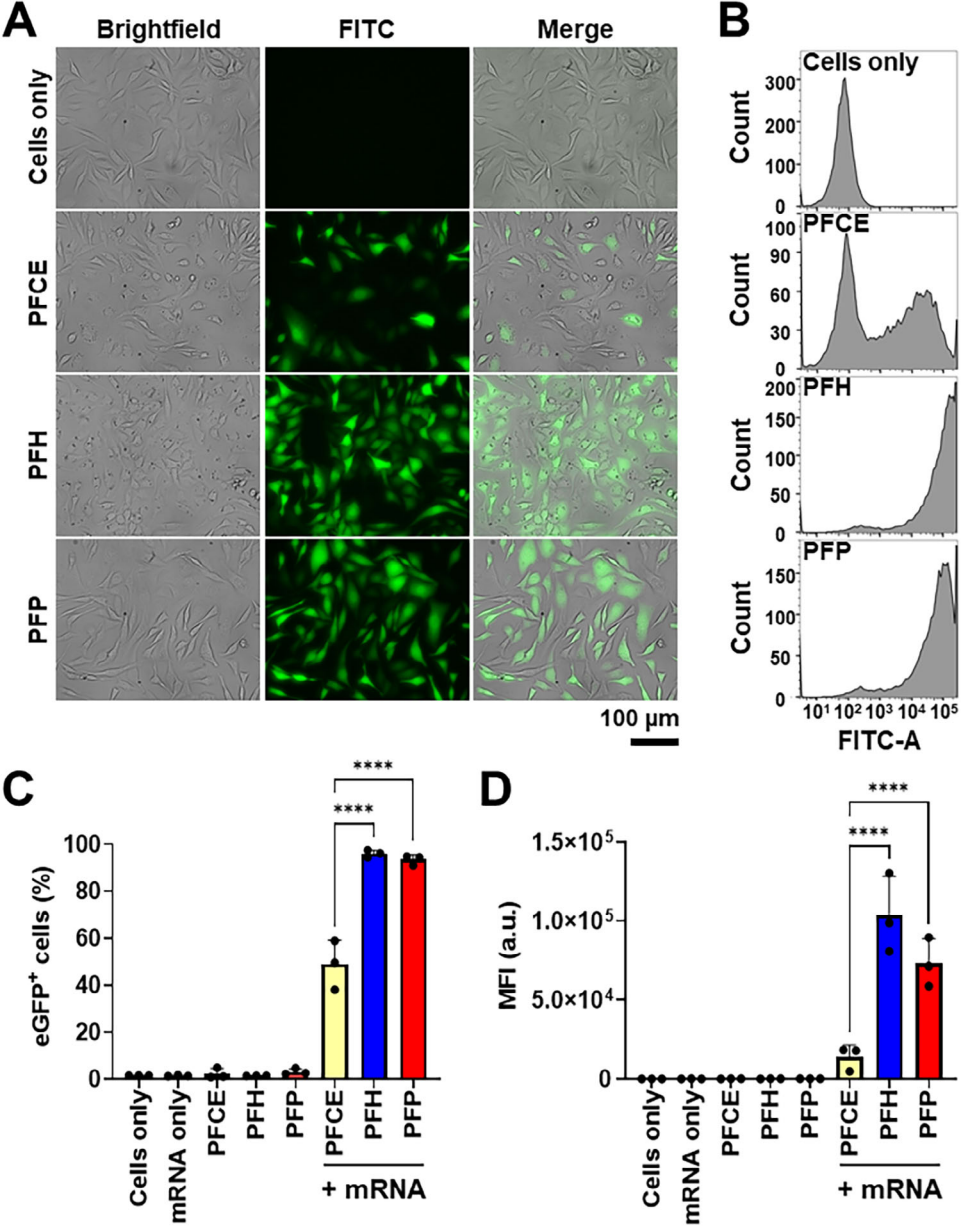
Transfection efficiency of PFCE, PFH, and PFP NDs via the direct transfection method after a 24‐hour incubation period. A) Representative photomicrographs showing the brightfield, eGFP fluorescence signals, and merged images of CHO cells. Scale bar = 100 µm. Flow cytometry data showing B) histograms of FITC‐A signals shifting right as the eGFP signal increases, C) the percentage of eGFP‐positive cells, and D) mean fluorescence intensity (MFI). Bar graphs are shown as mean ± SD from three independent experiments (n = 3); ns = not significantly different, ****p < 0.0001 by one‐way ANOVA with Tukey's multiple comparisons between all groups.

Brightfield microscopy images of CHO cells treated with eGFP mRNA‐loaded PFCE and PFH NDs (**Figure** **4**A) showed black pixels corresponding to droplets on the cell surface, while PFP‐treated cells showed no such features. These dark pixels are not visualized, possibly indicating the complete conversion of PFP into the gas phase during the incubation period at 37 °C. FITC channel (detected emission wavelength range of 475–650 nm) images in Figure 4A revealed higher expression of eGFP in PFH and PFP NDs‐treated cells, while PFCE ND‐treated cells exhibited lower expression.

Quantitative data on the transfection efficiency were confirmed using flow cytometry. Based on the results, distinct rightward FITC fluorescent shifts were observed with eGFP mRNA transfection compared with the cell‐only control group, especially PFH and PFP groups (Figure 4B), implying the effectiveness of ND‐mediated transfection. A count of cells expressing eGFP (eGFP‐positive cells) and mean fluorescence intensity (MFI) was also identified from flow cytometry. No eGFP expression was observed in negative controls using PFC NDs only without mRNA or mRNA alone. PFH and PFP NDs achieved over 90% eGFP‐positive cells, whereas PFCE‐treated cells reached only ≈50% (Figure 4C). Furthermore, the observed mean fluorescence intensities of PFH and PFP ND‐transfected cells are more than five times higher than those transfected with PFCE NDs (Figure 4D). This indicates that PFH and PFP NDs not only transfect more cells but also induce much more eGFP expression. In the direct transfection assay, the enhanced mRNA expression observed with PFH and PFP NDs likely stems from their higher volatility and thermal expansion rather than active endosomal escape. The mild vaporization of these low‐boiling point PFC cores under incubation conditions can generate transient physical stress on cellular membranes,^[^ ^44^ ^]^ facilitating the release of internalized mRNA into the cytosol.

Comparative analysis with nanoparticles having identical lipid shells showed significantly higher transfection efficiency for PFH NDs (Figure S4, Supporting Information), demonstrating the essential role of PFC core. Replacing DC‐cholesterol with cholesterol, 1,2‐dioleoyl‐3‐trimethylammonium‐propane (DOTAP), and 1,2‐dioctadecenyl‐3‐trimethylammonium propane (DOTMA) further confirmed DC‐cholesterol as the optimal component for effective mRNA delivery (Figure S4, Supporting Information). No eGFP expression was observed using cholesterol‐containing PFH NDs, which demonstrated the importance of cationic lipids for transfection. Furthermore, the PFH NDs formed with DC‐cholesterol showed a significantly higher percentage of cells expressing eGFP and increased mean fluorescence intensity, as compared to those formulated with DOTAP or DOTMA.

#### 2.4.2 Ultrasound‐Mediated Transfection

The impact of ultrasonic stimulation on the PFC ND‐mediated transfection of mRNA was investigated using CHO cells via insonation using burst mode (25 consecutive bursts per area at 100% power). While direct transfection establishes the efficacy of PFC NDs as an mRNA delivery system (Section 2.4.1), this method does not replicate physiological conditions, such as blood flow dynamics and transient tissue exposure to circulating mRNA delivery systems. To address these, PFC NDs were provided immediately before and promptly removed after treatment, simulating brief contact akin to in vivo exposure.

**Figure 5:**
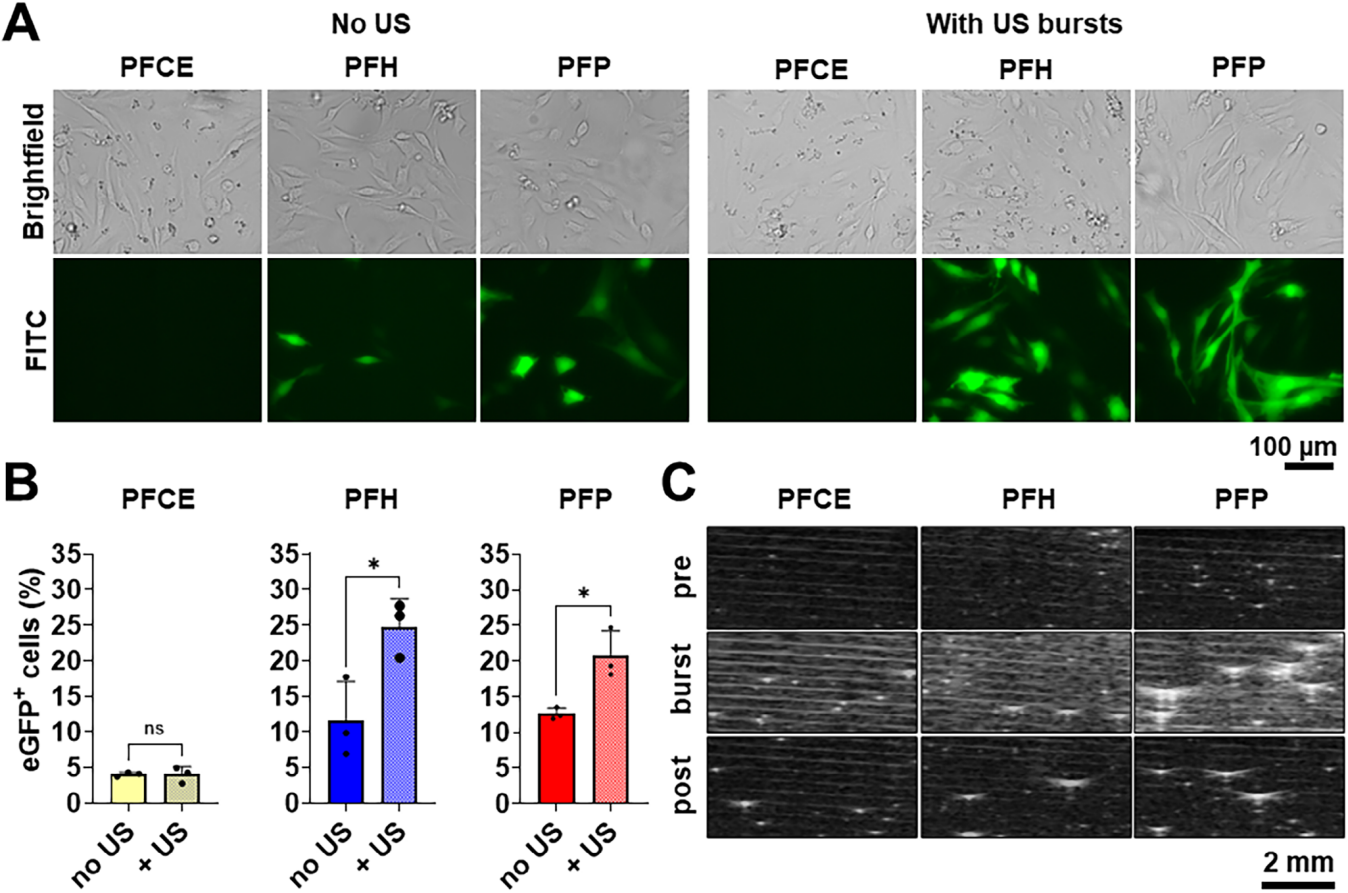
A) Representative photomicrographs showing the brightfield and eGFP fluorescence signals in CHO cells, 24 h post‐transfection with PFCE, PFH, and PFP NDs, either without or subjected to ultrasound (US) stimulation. Scale bar = 100 µm. B) Transfection efficiency of PFCE, PFH, and PFP NDs via the US‐stimulated transfection method after a 24‐h incubation period, represented by percentage of eGFP‐positive cells, measured by flow cytometry. CHO cells were treated with 5 µL PFC NDs + 0.5 µg eGFP mRNA with and without US exposure. Bar graphs are shown as mean ± SD from three independent experiments (*n* = 3); ns = not significantly different, *p < 0.05, **p < 0.01 by two‐way ANOVA with Tukey's multiple comparisons between all groups. C) B‐mode ultrasonograms of PFC ND dispersions before (pre), during (burst), and immediately after (post) exposure to burst ultrasound. Microbubble formation is indicated by the transformation of small grey pixels into large, bright, curved, to dragonfly‐shaped objects. Scale bar = 2 mm.

FITC channel photomicrographs in **Figure** **5**A and flow cytometry data in Figure 5B revealed significantly higher eGFP expression in CHO cells treated with mRNA‐loaded PFH and PFP NDs, in combination with ultrasound bursts, compared to the non‐insonated treatments. Similar to direct transfection results (Figure 4), cells treated with mRNA‐loaded PFCE NDs exhibit minimal to no eGFP expression, even with the application of burst ultrasound.

The observed transfection efficiency appears to correlate with ultrasound‐induced phase transitions captured in B‐mode ultrasonograms (Figure 5C). At low power (10%), pre‐burst imaging showed PFC NDs as small white specks against the black background. During ultrasound burst treatment (100% power), PFH and PFP NDs underwent significant phase transitions into microbubbles, appearing as larger and more reflective structures due to their increased surface area and acoustic response. Notably, PFP NDs exhibited a distinct dragonfly‐like morphology, reflecting their unique vaporization dynamics and stability as large microbubbles. The distinct “dragonfly‐like” ultrasound pattern observed for PFP microbubbles represents a scattering artefact from stable oscillation rather than structural deformation or vascular damage. Post‐burst ultrasonograms (10% power for imaging) further confirmed that the microbubbles formed from PFH and PFP NDs remained stable and dispersed in the medium, reinforcing their role in boosting mRNA transfection through inertial and stable cavitation mechanisms. In contrast, PFCE NDs showed minimal changes, indicating limited acoustic responsiveness and vaporization under these conditions. Cells exposed to mRNA‐loaded PFCE NDs also exhibited minimal transfection enhancement post insonation.

Ultrasound‐induced cavitation occurs in two forms: inertial and stable cavitation. In inertial cavitation, high‐intensity ultrasound causes rapid bubble expansion and collapse, generating localized shear forces, shock waves, and transient pores on cell membranes (sonoporation) that enhance genetic material and drug carrier penetration and uptake.^[^ ^14^, ^16^, ^45^ ^]^ Stable cavitation, induced by low‐intensity ultrasound, involves bubble oscillation that rearranges cell membranes and promotes or even bypasses endocytosis of mRNA‐loaded droplets.^[^ ^46^, ^47^ ^]^ The enhanced transfection observed for PFH and PFP NDs underscores the role of acoustic forces, acoustic vaporization, and subsequent cavitation, along with their associated biomechanical effects, in improving mRNA delivery efficiency.

These results align with previous studies demonstrating ultrasound‐mediated genetic material uptake using PFC NDs.^[^ ^48^ ^]^ Despite brief exposure (≈10 s), resembling vascular conditions, significant mRNA transfection efficiency was achieved, strongly supporting the feasibility of translating this technology into in vivo applications, especially given the widespread availability of ultrasound equipment in clinical settings.

### 2.5 In Vivo Biodistribution of PFC NDs

**Figure 6:**
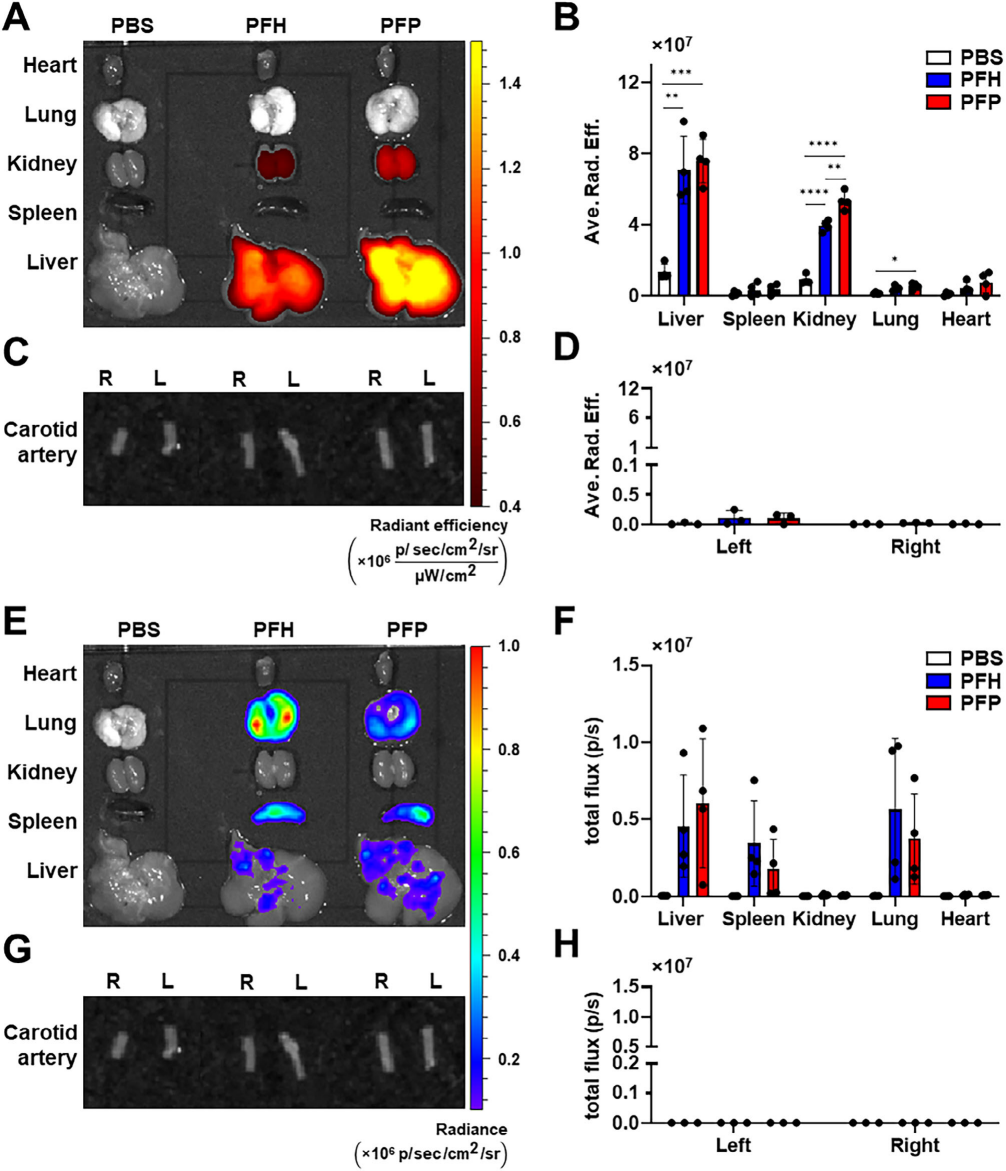
Biodistribution and bioluminescence imaging of Cy7‐labeled PFH and PFP NDs, loaded with Nanoluc mRNA. A–D) IVIS imaging of C57BL/6 mice 24 h post intravenous administration of PFH and PFP NDs. Representative fluorescence images of (A) major organs and (C) carotid arteries (*R* – right and *L* – left). (B,D) Quantification of Cy7 fluorescence as average radiant efficiency (ave. rad. eff.) [p/s/cm^2^/sr] / [µW/cm^2^]. E–H) Bioluminescence imaging showing the fluorofurimazine–NanoLuc reaction, confirming successful mRNA translation in the corresponding organs and tissues. Representative bioluminescence images of (E) major organs and (G) carotid arteries. F,H) Quantification of bioluminescence as total flux (p/s). Color scales indicated Cy7 fluorescence (A,C) and bioluminescence (E,G) signal intensities. Data represent mean ± SD (n > 3). One‐way ANOVA (major organs: B,F) with Tukey's multiple comparisons between all treatment groups per organ: **p < 0.01, ***p < 0.001, ****p < 0.0001. Two‐way ANOVA (carotid arteries: D,H) with Tukey's post hoc test comparing all means showed no significant differences among groups.

After confirming the in vitro transfection efficiency and ultrasound‐enhanced mRNA delivery, we next examined the in vivo biodistribution and mRNA expression of a bioluminescence protein to further validate their biosafety and suitability for controlled mRNA delivery. Cy7‐labeled PFH and PFP NDs (3 mg mL^−1^) loaded with Nanoluc mRNA (7.5 µg) were injected intravenously. After 24 h, major organs and carotid arteries were collected for Cy7 fluorescence and Nanoluc–fluorofurimazine bioluminescence imaging using the In Vivo Imaging System (IVIS). Cy7 fluorescence signals were predominantly detected in the liver and kidneys, indicating hepatic and renal clearance of PFC NDs. Bioluminescence signals, reflecting Nanoluc expression, were observed mainly in the liver, spleen, and lungs, confirming that the NDs successfully delivered, released mRNA, and translated the protein in vivo. Based on the results shown in **Figure** **6**, the presence of Cy7 fluorescence without corresponding bioluminescence in the kidneys suggests passive elimination of fluorescent lipid metabolites rather than active transfection, consistent with the expected excretory pathway for amphiphilic dyes within 24 h post‐injection.

The colocalization of Cy7 and bioluminescence in the liver reflects both accumulation and translation, in line with the known hepatic tropism of nanoparticle systems mediated by Kupffer cell phagocytosis and ApoE‐dependent hepatocyte internalization.^[^ ^49^ ^]^ In the spleen, bioluminescence was observed despite low Cy7 fluorescence, suggesting rapid uptake and disassembly of NDs by splenic macrophages, leading to mRNA release prior to fluorophore clearance. The spleen localization likely results from nanoparticle uptake by the reticuloendothelial sinusoids (200–500 nm), consistent with the spleen's physiological role in filtering particles larger than ≈200 nm.^[^ ^50^ ^]^ Meanwhile, the strong bioluminescence in the lungs likely arises from a cationic DC‐cholesterol component that promotes pulmonary deposition through protein corona‐mediated interactions.^[^ ^51^, ^52^ ^]^

Both PFH and PFP formulations exhibited comparable biodistribution patterns, though PFH NDs produced more consistent signal intensity. Minimal fluorescence and bioluminescence in the carotid arteries confirmed low baseline transfectability under non‐ultrasound conditions, establishing a suitable control for subsequent ultrasound‐triggered delivery experiments (Section 2.6).

### 2.6 In Vivo Ultrasound‐Mediated Transfection of the Vasculature

**Figure 7:**
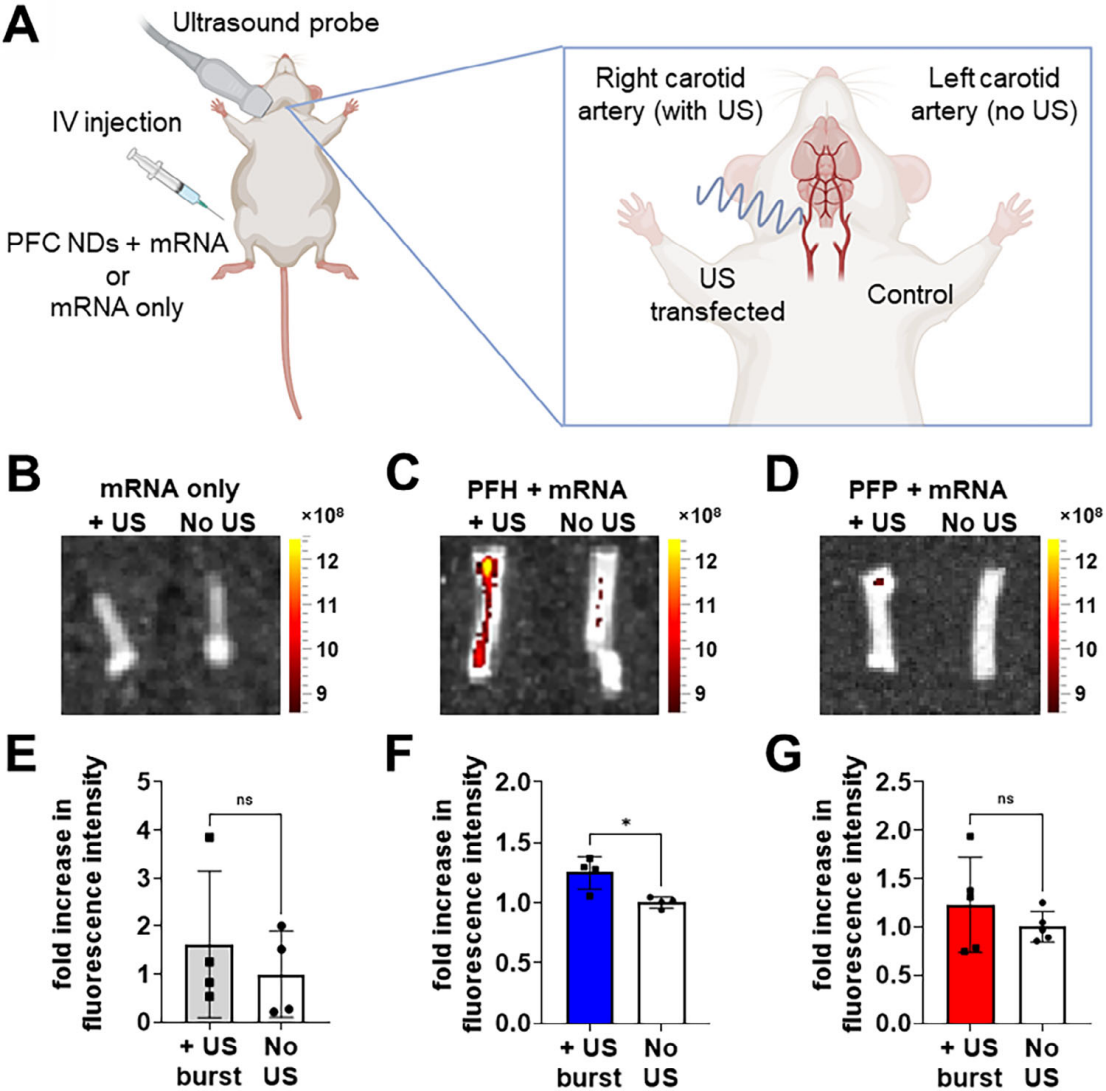
A) Schematic diagram showing ultrasound‐mediated delivery of mCherry mRNA‐carrying PFC NDs to the right carotid artery of a mouse (created with BioRender.com). Left carotid arteries were used as “no US” controls. Fluorescence images of isolated mouse carotid arteries 24 h post‐treatment with B) mCherry mRNA only (control), C) PFH NDs, and D) PFP NDs with mCherry mRNA without ultrasound (US) (left carotid artery) and with US burst treatment (right carotid artery). Color scale shows the fluorescence signal intensities as average radiant efficiency in [p/s/cm^2^/sr] / [µW/cm^2^]. Bar graphs showing the quantitative analysis using average radiant efficiency in fluorescence photomicrographs of corresponding isolated carotid arteries 24 h post‐treatment with E) mCherry mRNA only, F) PFH NDs, and G) PFP NDs. Bar graphs are shown as mean ± SD from independent animals (n ≤ 5); ns = not significantly different, *p < 0.05 by two‐tailed, paired *t*‐test, comparing non‐US and US burst‐treated samples.

Building on the successful in vitro imaging and transfections, as well as in vivo biodistribution results, we further explored the potential application and efficacy of mCherry mRNA loaded PFH and PFP NDs as ultrasound‐triggered mRNA delivery systems in vivo (**Figure** **7**A). PFCE NDs were excluded in this in vivo study due to their suboptimal performance demonstrated in Section 2.4. Each mouse has two carotid arteries: the right artery was exposed to ultrasound burst, while the left served as an internal control. Using a femoral vein catheter, a bolus of mCherry mRNA‐loaded PFH or PFP NDs, was injected into the mice. To determine the critical role of PFC NDs in ultrasound‐mediated mRNA delivery, an equivalent dose of naked mCherry mRNA (without PFC NDs) was administered together with ultrasound bursts as a control. This control was designed to exclude the possibility that ultrasound alone could facilitate mRNA uptake through sonoporation or acoustic cavitation effects. The use of a catheter ensured the integrity of the PFC NDs injected, as the stress and shear forces typically introduced by manual injection can compromise their stability and effectiveness. Upon first observation of PFC NDs within the cardiac region, we performed ultrasound burst stimulations on the right carotid arteries (Figure S5, Supporting Information). Both carotid arteries were excised after 1 h and cultured overnight in DMEM at 37 °C to allow for mCherry expression.

Figure 7B–D show representative fluorescence images overlaid with visible light photographs of the excised carotid arteries, providing structural alignment for the observed fluorescence signals in ultrasound‐treated right carotid arteries of mice injected with naked mCherry mRNA (“mRNA only” group), mRNA‐loaded PFH or PFP NDs. mCherry mRNA was selected for this in vivo study due to its strong and stable fluorescence signal, which offers greater tissue penetration and visibility compared to eGFP, while also minimizing interference from tissue autofluorescence, thereby ensuring reliable detection of transfection in excised vascular tissues.^[^ ^53^ ^]^ Minimal fluorescence was observed in both the “mRNA only” control groups (with and without ultrasound burst), as well as in non‐insonated left carotid arteries of PFH‐ and PFP‐treated mice, indicating that ultrasound exposure alone was insufficient for detectable mRNA transfection. Quantitative analysis (Figure 7E–G) confirmed that ultrasound bursts did not significantly enhance mCherry expression without the presence of PFC NDs. In contrast, PFH NDs with ultrasound burst induced a significant increase in fluorescence intensity compared to non‐US‐treated arteries (*p* < 0.05). While PFP NDs also induced mCherry expression, high signal variability rendered the difference statistically insignificant.

The differences in mCherry expression between PFH and PFP NDs can be attributed to their distinct phase transition behaviors at physiological temperatures. PFH NDs remained stable and underwent controlled vaporization only upon ultrasound exposure, allowing uniform activation and reproducible mRNA delivery. In contrast, the low boiling point and high volatility of PFP cause premature vaporization shortly after systemic injection, even in the absence of ultrasound, resulting in transfection outcomes and greater inter‐animal variability. Conversely, the greater thermal stability of PFH NDs ensures controlled vaporization only under acoustic exposure, resulting in more consistent and reproducible mRNA delivery and fluorescence enhancement.

A key challenge in vascular mRNA transfection is achieving effective margination of NDs toward vessel walls under dynamic blood flow. The high density of PFC NDs enhances their ability to marginate, while ultrasound application further facilitates their movement toward the endothelium through acoustic radiation forces and acoustic microstreaming effects.^[^ ^54^, ^55^ ^]^ However, PFP NDs vaporize too readily, forming buoyant microbubble clusters that not only block further ND attachment but also fail to distribute effectively to the lower vessel regions. Their larger size and buoyancy also increase the likelihood of being carried away by blood flow, reducing their effectiveness in targeting the vessel walls. PFCE NDs, although stable, show limited ultrasound responsiveness, reducing their effectiveness for mRNA delivery by not generating sufficient cavitation or endosomal escape. PFH NDs overcome these challenges by providing optimal stability and responsiveness at physiological temperatures, maintaining uniform dispersion, and facilitating efficient endothelial interaction across the vessel lumen. These properties make PFH NDs a promising candidate for precise and effective vascular mRNA delivery, addressing the key limitations observed with other PFC NDs.

These findings highlight the translational potential of PFH NDs as a robust platform for ultrasound‐mediated mRNA delivery in cardiovascular applications. Their ability to achieve high transfection efficiency stems from their optimal balance between nanomaterial stability and acoustic responsiveness, enabling precise endothelial targeting under dynamic physiological conditions. Furthermore, PFH NDs demonstrate consistent performance across multiple experimental setups, including in vitro and in vivo models, which strengthens their viability for clinical applications. By minimizing the challenges associated with blood flow dynamics and ensuring reliable delivery, PFH NDs offer a promising solution for advancing mRNA‐based therapies in cardiovascular medicine.

### 2.7 In Vivo Safety Profile of PFC NDs

The biosafety of the PFC NDs was comprehensively evaluated using physiological, hematological, and biochemical indicators obtained from the in vivo biodistribution and mRNA transfection studies.

**Figure 8:**
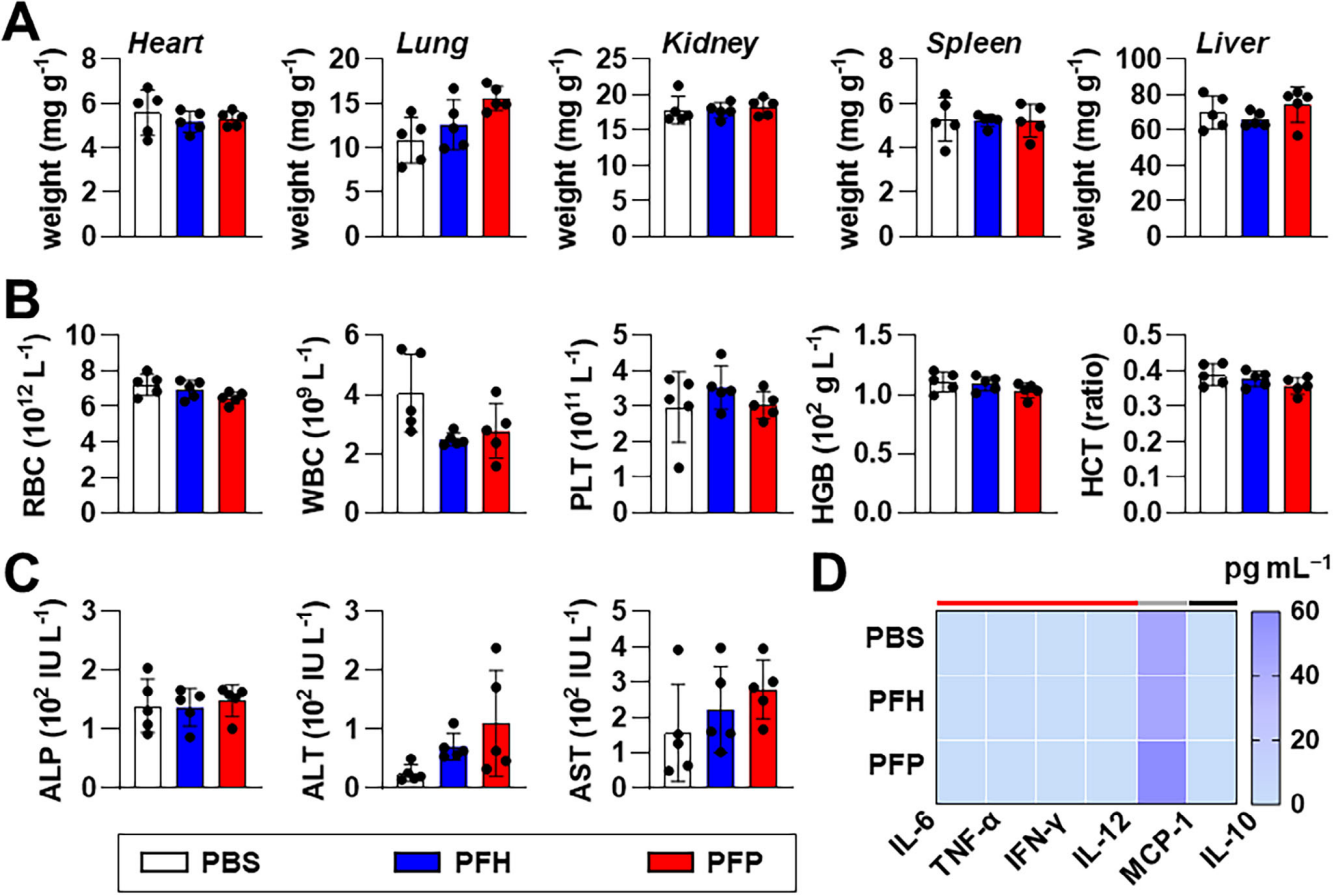
Biological safety of PFH and PFP NDs. A) Body weight‐normalized organ weights, B) Sysmex complete blood count data, C) liver function markers, and D) inflammatory cytokine expression in plasma of C57BL/6 mice, collected 24 h post‐administration (IV) of mRNA‐loaded PFH and PFP NDs. Heat map cells in (D) are classified by cytokine function: pro‐inflammatory (red), chemokine (grey), and anti‐inflammatory (black). Bar graphs (A–C) are shown as mean ± SD from independent animals (*n* = 5). All datasets were analyzed using one‐way ANOVA with Tukey's post hoc test (comparing all means), except for ALP in (C), which was analyzed using Kruskal–Wallis test with Dunn's multiple comparison test (comparing all means); no significant differences were found among groups.

Major organs, including the heart, liver, spleen, lungs, and kidneys, were weighed as organ mass serves as a sensitive marker of systemic toxicity and pathological stress.^[^ ^56^ ^]^ As shown in **Figure** **8**A, no statistically significant differences in organ weights were observed between PFH‐ or PFP‐treated mice and PBS control group. These findings indicate that the PFC NDs did not induce toxicity, inflammation, or organ hypertrophy, particularly in the liver and spleen (organs typically affected by nanoparticle‐induced stress or accumulation).

Complete blood count (Sysmex) analysis revealed no detectable changes in red or white blood cell counts, platelet levels, hemoglobin concentration, or hematocrit values (Figure 8B), confirming the excellent hemocompatibility of both PFH and PFP NDs.

Given the high mRNA expression observed in the liver and spleen during the biodistribution study, serum levels of hepatic function biomarkers were further analyzed (Figure 8C). The three key liver enzymes, alkaline phosphatase (ALP), alanine transaminase (ALT), and aspartate transaminase (AST), remained within physiological ranges across all groups. Although a slight elevation in ALT and AST levels was detected in mice treated with PFC NDs compared to PBS control, the differences were not statistically significant, indicating no evidence of hepatocellular injury.

Cytokine profiling (Figure 8D; Figure S6, Supporting Information) further demonstrated a favorable immunological safety profile. Treatment with PFH and PFP NDs did not significantly alter the plasma levels of IL‐6, TNF‐α, IFN‐γ, IL‐12, or IL‐10. A modest increase in MCP‐1 secretion was noted but remained within the normal physiological range,^[^ ^57^ ^]^ reflecting minimal immunogenicity.

Overall, these findings demonstrate the favorable biosafety profile of PFC NDs, supporting their suitability for in vivo mRNA delivery and ultrasound‐guided theranostic applications.

## 3 Conclusion

This study details a comprehensive exploration of PFC NDs as an innovative platform for ultrasound‐mediated mRNA delivery. Through diligent development and characterization, we demonstrated the unique capabilities of PFCE, PFH, and PFP NDs for both imaging and therapeutic applications. These NDs, with diameters ranging from 200 to 300 nm and positive surface charges, facilitate efficient mRNA capture and transfection. In vitro experiments confirmed their acoustic contrast enhancement in tissue‐mimicking phantoms and the stability‐dependent transfection efficiency of eGFP mRNA into CHO cells. In vitro studies confirmed their strong acoustic contrast in tissue‐mimicking phantoms and demonstrated that the fluorinated cores enabled ^19^F MRI detection with distinct chemical shifts, allowing simultaneous discrimination of multiple PFC compositions. More interestingly, we noted that PFH and PFP NDs, in particular, exhibited superior responsiveness to ultrasound, leading to enhanced mRNA transfection compared to PFCE NDs. Bio‐ and hemocompatibility assessments confirmed the safety of all three NDs, indicating their suitability for clinical applications with a wide range of tolerable doses. No significant cytotoxicity or adverse effects were observed in our studies, reinforcing their potential for safe therapeutic use.

In vivo biosafety and biodistribution analyses confirmed the minimal toxicity of all tested formulations and their predominant hepatic and renal clearance. In vivo imaging using Cy7‐labeled PFC NDs and Nanoluc mRNA revealed complementary biodistribution and expression profiles, where Cy7 fluorescence was mainly detected in the liver and kidneys, consistent with clearance pathways, while NanoLuc bioluminescence indicated active mRNA expression primarily in the liver, spleen, and lungs. No detectable fluorescence or bioluminescence was observed in the carotid arteries. Controlled release experiments using mCherry mRNA further validated the translational feasibility of PFH NDs for ultrasound‐triggered vascular delivery to murine carotid arteries. PFH NDs achieved the most consistent and efficient transfection, while PFP NDs exhibited greater variability due to excessive vaporization and microbubble clustering. This confirms that while PFH NDs distribute systemically following intravenous administration, mRNA expression remains strictly localised to the insonated region. The observed signal in the ultrasound‐treated carotid artery therefore arises from spatially confined vaporization and payload release, supporting the platform's capacity for precise, on‐demand gene delivery.

In summary, the three PFCs examined exhibit distinct physical and acoustic properties, validating their comparative assessment. PFH NDs showed the most favorable profile for ultrasound‐based theranostics, combining acoustic stability, efficient ultrasound‐triggered mRNA release, and consistent in vivo protein expression. PFP NDs, due to their low boiling point and high volatility, exhibited inconsistent behavior and limited translational potential. PFCE NDs were the most thermally stable and best suited for ^19^F MRI detectability, but required more prolonged incubation owing to slower release kinetics. Among these, PFH NDs emerge as the lead candidate for next‐generation ultrasound‐controlled theranostic platforms, offering a powerful and adaptable foundation for advancing mRNA‐based therapies.

The present work focused on establishing the feasibility, biosafety, and controllable delivery performance of this ultrasound‐responsive mRNA platform. Future studies involving diverse cardiovascular murine models and promising mRNA candidates targeting inflammation, vascular repair, and thrombosis prevention will be used to further validate this platform.

## 4 Experimental Section

### Materials and Samples

The following chemicals and solvents were used as received: perfluoro‐15‐crown‐5‐ether (PFCE, C_15_F_30_O_5_, abcr GmbH), perfluorohexane (PFH, C_6_F_14_, FluoroChem), perfluoropentane (PFP, C_5_F_12_, Synquest Laboratories), 3‐(4,5‐dimethylthiazol‐2‐yl)‐2,5‐diphenyltetrazolium bromide (MTT, C_18_H_17_N_5_S, Invitrogen), dimethyl sulfoxide (DMSO, C_2_H_6_OS, Sigma‐Aldrich), and chloroform (CHCl_3_, Sigma‐Aldrich). *N*1‐methyl‐pseudouridine‐modified mRNA coding for the enhanced green fluorescent protein (eGFP mRNA, 1187 nt) and *N*1‐pseudo pseudouridine‐modified mRNA coding for the mCherry protein (981 nt) were purchased from BASE mRNA Facility at The University of Queensland. Cell culture media and components were supplied by ThermoFisher Scientific: Dulbecco's Modified Eagle Medium (DMEM, high glucose, Gibco) was either used without further processing (FBS‐free medium) or supplemented with 1% *L*‐glutamine, 1% streptomycin/penicillin, and fetal bovine serum (FBS) for cell culture.

### PFC ND Fabrication and mRNA Loading

A thin film–hydration method was adopted for the fabrication of PFC emulsion NDs. Different quantities of lipid stock solutions (Table S1, Supporting Information) were mixed in 5 mL glass vials, which were then placed in a vacuum evaporator (PC 3001 VARIOpro) to completely evaporate the organic solvent. PBS (950 µL) and PFC (50 µL, final concentration of 5% (v/v) PFCE, PFH, or PFP) were pipetted into the vials with the dehydrated lipid films, followed by microtip sonication (10 min total sonication time, 50% amplitude, sonication cycle 10 s ON and 10 s OFF) using a QSonica Q700 sonicator. The resulting dispersions were then injected into a Lv1 benchtop microfluidizer homogenizer (Microfluidics, USA) at 18 000 kPa for 15 cycles. ND dispersions were stored in screw‐capped vials at 4 °C prior to use. To load eGFP mRNAs, NDs were incubated with mRNA for 20 minutes to allow interaction between mRNA and NDs.

### DLS

Droplet sizes and ζ‐potentials of the fabricated NDs (10 µL ND dispersion diluted with 990 µL water) were measured by dynamic light scattering (DLS) and electrophoretic light scattering (ELS), respectively, using a Malvern Zetasizer (Malvern Instruments Ltd., Worcestershire, United Kingdom) spectrometer (wavelength λ = 632.8 nm).

### mRNA Entrapment Efficiency by PFC NDs

The ability of PFC NDs to capture mRNA through electrostatic adsorption was evaluated using a modified RiboGreen assay.^[^ ^58^ ^]^ Briefly, PFC ND dispersion (50 µL) was mixed with mCherry mRNA (2.5 µg, 0.8 µL) and incubated on ice for 15 min to allow complexation. In parallel, 50 µL PBS containing the same amount of mCherry mRNA served as the 100% mRNA control, while PFC NDs (50 µL) mixed with 0.8 µL of PBS were prepared to account for matrix effects (ND control). Following incubation, all samples were centrifuged at 15 000 ×*g* for 30 s at 4 °C. Supernatants (20 µL) from each dispersion (or from the upper fraction of the mRNA only control) were carefully collected. A 2 µL aliquot from each supernatant was analyzed using the RiboGreen assay according to the manufacturer's protocol. The mRNA entrapment efficiency (EE) was calculated using Equation (1).

(1) $$EE=\left(1-\frac{Sample\;FI-ND\;control\;FI}{mRNA\;control\;FI}\right)\times 100\%$$

### TEM

Emulsion samples (5 µL) were pipetted onto a Parafilm‐covered glass plate. Carbon‐coated 300 mesh copper grids were incubated for five minutes on top of the emulsion droplets with the carbon face down. Following incubation, the excess emulsion sample was blotted away with filter paper, and the grids were placed face down onto 5 µL droplets of 1% aqueous uranyl acetate. The grids were incubated on uranyl acetate for 30 seconds, after which the uranyl acetate was blotted away with filter paper. This staining and blotting step was repeated two more times, before drying the grids for about 5 to 10 min. Imaging was performed immediately after preparation under low‐dose transmission electron microscopy conditions (vacuum, 80 keV) using a JEOL JEM‐1400 Plus (JEOL Ltd., USA).^[^ ^59^ ^]^

### Observation of Bubble Formation by Optical Microscopy

Photomicrographs of bubble formation induced by an external heat source were obtained using a CCD camera (Flea3, Point Grey, Richmond, BC, Canada) coupled to an Eclipse Ci‐S light microscope (Nikon Instruments, Inc., USA). Temperature control was achieved using a Linkam Scientific PE120 Peltier temperature stage, coupled to a recirculating water bath, with an accuracy of ± 0.1 °C, at a heating rate of 2.5 °C min^−1^.^[^ ^33^, ^35^ ^]^

### Thermogravimetric Analysis

TGA was performed on a Mettler Toledo TGA‐DSC 1 STARe System using standard 100 µL aluminium metal pans over a temperature range of 25–90 °C under nitrogen gas flow (30 mL min^−1^), at a heating rate of 3 °C min^−1^.^[^ ^34^ ^]^

### Ultrasound Imaging

A Vevo 2100 high‐resolution imaging system (VisualSonics Inc., Toronto, Canada), equipped with its 22–55 MHz MS 550D transducer (operating frequency of 40 MHz), was used for both in vitro and in vivo experiments. Imaging was conducted with a duty cycle of 100% in non‐linear contrast (NLC) mode, which simultaneously provides a diagnostic B‐mode view and a contrast imaging view at 6–10% transmit power. Ultrasound bursts are delivered at 100% transmit power. The methods^[^ ^59^ ^]^ below were used in imaging PFC NDs in tissue‐mimicking phantoms and in live mice.

*In Vitro Imaging Using Tissue‐Mimicking Gel Phantoms*: Ultrasonograms were obtained from tissue‐mimicking phantoms made from 2% agarose hydrogels, where 100 µL of PFC NDs (5% PFC) were loaded into the empty wells. PBS was used as a control, with three replicates per condition (n = 3).

*In Vivo Imaging in Mouse*: All animal experiments involving ultrasound imaging were approved by the Alfred Medical Research and Education Precinct Animal Ethics Committee (approval E/8335/2022/B). C57BL/6 mice (20–30 g, provided by the Alfred Medical Research and Education Precinct (AMREP) Animal Services) were injected intraperitoneally with ketamine (100 mg kg^−1^) and xylazine (5 mg kg^−1^). The abdominal area was shaved to allow imaging of the inferior vena cava. A small incision was made on the thigh of the mouse, and a catheter was inserted and secured into the femoral vein. The mouse was positioned supine on a VisualSonics imaging station. Either PBS (control) or ND dispersions (100 µL) were administered via the catheter.

### Magnetic Resonance Imaging

Data were acquired at a Bruker AVANCE III 9.4T wide‐bore NMR spectrometer driven by ParaVision 5.1 (Bruker, Rheinstetten, Germany) and operating at 400.13 and 376.54 MHz for ^1^H and ^19^F measurements, respectively.^[^ ^21^ ^]^ Imaging was conducted using a Bruker Micro 2.5 microimaging unit with actively shielded gradients (1.5 T m^−1^) and a 25 mm birdcage resonator tunable to both ^1^H and ^19^F. A PFC ND cocktail, comprised of 5% (v/v) PFCs from equal volumes of the three PFC ND dispersions, was imaged using a 2D ^19^F chemical shift imaging sequence as previously described.^[^ ^60^ ^]^

### In Vitro Transfection

*Cell Culture*: Chinese Hamster ovary (CHO) cells were cultured in DMEM with 10% (v/v) FBS and 1% L‐glutamine, and 1% streptomycin/penicillin at humidified conditions (37 °C) with 5% CO_2_ supply using Steri‐cycle CO2 Incubator (Thermo Fisher Scientific, Germany). Prior to transfection, ≈1 × 10^6^ cells were seeded into each sterile Petri dish (35 mm × 15 mm, Falcon). Once a 70–80% confluency has been reached, the old medium was removed, followed by washing with sterile phosphate buffer saline (100 mM PBS, pH 7.4), and sample treatment for transfection.

*Direct Transfection*: Cells were added with 1 mL transfection medium (DMEM with 1% *L*‐glutamine and 1% streptomycin/penicillin), containing 5 µL PFC NDs with 0.5 µg eGFP mRNA, followed by incubation for 24 h at humidified conditions (37 °C) with 5% CO_2_ supply using Steri‐cycle CO_2_ Incubator.

*Ultrasound‐Mediated Transfection in CHO Cells*: Cells were prepared as mentioned in the sub‐sections Cell Culture and Direct Transfection under the section In Vitro Transfection. To simulate bloodstream dilution and ensure sufficient volume for ultrasound penetration, an additional 6 mL of fresh sample‐free medium was added after the initial treatment. For ultrasound exposure, the MS 550D transducer was sterilized and partially submerged in the medium. A single ultrasound burst (100% transmit power) was delivered every 1.5 mm across the petri dish, covering 25 exposure regions with a total of 25 bursts. Immediately after ultrasound exposure, the medium was aspirated, and cells were gently washed with PBS to remove the unbound PFC NDs, mimicking circulatory clearance. As a control, sets of samples underwent the same procedure without ultrasound exposure, but included an additional 5‐minute incubation period to match the exposure time of the ultrasound‐treated cells. Finally, washed petri dishes containing the cells were replenished with 1 mL DMEM and incubated for 24 h.

### Fluorescence Microscopy and Flow Cytometry

To qualitatively confirm transfection via the expression of eGFP on transfected cells, cells treated with the samples (and control cells) were imaged using an IX81 Olympus microscope (Olympus, Tokyo, Japan) in brightfield mode and via the FITC fluorescence channels (exposure time = 2 s). To accurately determine transfection efficiency via eGFP‐positive cell counts, cells were analyzed using a LSRFortessa X‐20 flow cytometer and FlowJo software (BD Biosciences, USA).

### MTT Assay

Viability of CHO cells after exposure to PFC NDs was evaluated using a standard MTT protocol. Briefly, cells (1 × 10^5^ cells per well) were seeded into 96‐well plates in 100 µL volume with cell culture medium (DMEM supplemented with 10% (v/v) FBS, 1% (v/v) penicillin–streptomycin solution, and 1% (v/v) L‐glutamine) and incubated for 24 h, prior to treatment. Medium from each well was replaced with fresh medium containing different concentrations of PFC NDs (0.3, 1.5, 15, 150, and 300 µg mL^−1^), followed by incubation at 37 °C for 24 h. MTT solution (10 µL, 5 mg mL^−1^) was then added per well, followed by a 4‐h incubation period at 37 °C. After incubation, the medium was removed and replaced with 100 µL DMSO in each well to dissolve formazan crystals, followed by spectrophotometric reading at 570 nm using a microplate reader (BMG Labtech FLUOstar Omega Microplate Reader, Germany). Cell viability was calculated and normalized to 100% using the absorbance of wells with PBS‐treated cells.

### Hemocompatibility Assay

Human blood was collected in citrate tubes from healthy volunteers with informed consent, in compliance with the relevant laws and institutional guidelines, as approved by the Baker Heart and Diabetes Institute, Melbourne, Australia (Alfred Human Ethics, project number: 58/24). Increasing concentrations of the three different PFC ND formulations (0.3, 1.5, 15, 150, and 300 µg mL^−1^), PBS, or Triton X‐100 were added to human whole blood at a ratio of 1:20 (sample‐to‐blood ratio) and placed on an incubated shaker (150 rpm) at 37 °C for 1 h. Aliquots (20 µL) of the treated whole blood samples were taken for analysis using an automated cell counter (Sysmex XN‐550, Japan). The remaining blood samples were then centrifuged (2000 rpm, 10 min) to separate plasma from blood. Plasma was collected, and absorbance at 550 nm was measured using a 96‐well plate reader (BMG Labtech FLUOstar Omega Microplate Reader, Germany). Hemolysis was calculated and normalized to 100% hemolysis (Triton X‐100). During data normalization, occasional negative values (<0%) arising from minor instrumental variation were set to zero, as hemolysis cannot physiologically occur below 0%.

### Biodistribution and Biosafety

All experiments for in vivo biodistribution were approved by the Alfred Medical Research and Education Precinct Animal Ethics Committee (No. E/10630/2023/B). C57BL/6 mice (mixed‐sex, 18–25 g) were randomly assigned to receive 100 µL Cy7‐labeled PFC NDs loaded with 7.5 µg of Nanoluc mRNA or an equal volume of PBS via intravenous injection. At 24 h, mice were administered with 100 µL (0.46 µmol) Nano‐Glo Fluorofurimazine (FFz) substrate intraperitoneally, and after 15 min were euthanized (ketamine/xylazine, 300/50 mg kg^−1^). Blood was collected in Clexane via intracardiac puncture for Sysmex analysis, liver enzyme test, and inflammatory cytokine analysis. After perfusion with PBS, major organs (liver, spleen, kidney, lungs, heart) and carotid arteries were harvested and weighed, followed by imaging using an IVIS Lumina III imaging system (PerkinElmer, USA) for both Cy7 fluorescence and bioluminescence.

### Inflammatory Cytokine Quantification

The mouse serum was subsequently analyzed with BDTM Cytometric Bead Array (CBA). The Mouse Inflammation Kit (BD, 552364; Mulgrave, Australia) was used according to the manufacturer's instructions.

### In Vivo Ultrasound‐Mediated mRNA Delivery

All animal experiments involving ultrasound imaging were approved by the Alfred Medical Research and Education Precinct Animal Ethics Committee (No. E/8335/2022/B). Please see sub‐section In Vivo Imaging in Mouse under the section Ultrasound Imaging for the ultrasound machine set‐up and animal preparations. Shaving was performed on the neck of the mice (C57BL/6, mixed‐sex, 18–25 g). The right carotid artery was located using diagnostic B‐mode, before switching to NLC mode (10% transmit power) with the burst ultrasound function (100% transmit power at burst). Samples (150 µL PFH or PFP NDs with 7.5 µg mCherry mRNA) were administered via the catheter. Following the first detection of PFC NDs in the cardiac region, 2 ultrasound bursts were applied every 30 s over 5 min in the right carotid artery. The left carotid did not receive ultrasound stimulation; hence, it was used as an internal negative control. The mice were placed on heated mats for 1 h, before euthanasia by intraperitoneal injection of a combination of ketamine (300 mg kg^−1^) and xylazine (60 mg kg^−1^). Blood was collected via heart puncture into syringes containing enoxaparin sodium (Clexane, 0.4% v/v). Organs were then perfused with sterile PBS. Both carotid arteries were excised (the left non‐ultrasound stimulated artery was collected as a control) and placed into 1 mL DMEM with 10% (v/v) FBS and 1% L‐glutamine, and 1% streptomycin/penicillin in 24 well cell culture plates at 37 °C in humidified conditions using Steri‐cycle CO_2_ Incubator (Thermo Fisher Scientific, Germany). To ensure blinding, different personnel were assigned to distinct experimental tasks. One set of researchers performed the injections, while another set, blinded to the groups, conducted the imaging and subsequent data analysis.

Twenty‐four hours later, the carotid arteries were imaged using an IVIS Lumina III imaging system (PerkinElmer, USA) in two modes: visible‐light photography to provide an anatomical underlay and mCherry measurements using an excitation maximum of 580/20 nm and an emission filter of 620/40 nm. Regions of interest were defined by tracing the photographic underlay of the vessels using the Living Image analysis software (PerkinElmer) and the average radiant efficiency was measured. Fold increase in fluorescence intensity relative to the controls (left carotid artery with no US treatment) was calculated using the Equation (2).

(2) $$fold\;increase=\frac{observed\;radiant\;efficiency}{mean\;radiant\;efficiency\;in\;sample\;controls}\times 100\%\;$$

### Statistical Analysis

All data were assessed for normality using the Shapiro–Wilk test. Evaluation of outliers was determined using ROUT (Q = 10% for likely outliers). An F test was used to analyze equality of variance for data sets with two groups, whereas the Brown–Forsythe test was used for data sets with more than three groups. Data was reported as mean ± standard deviation (SD). The sample size (*n*) for each statistical analysis is clearly stated in each respective section (*n* ≥ 3). Statistical methods are as follows: for data sets of two groups with parametric data and equal variances, statistical analysis was performed using t test (two‐tailed, paired for the in vivo studies, and unpaired in other tests); and for data sets of more than two groups with parametric data and equal variances, one‐way ANOVA followed by post hoc analysis by Tukey test was used. In the event of unequal variance, the Brown‐Forsythe and Welch ANOVA with Dunnett T3 multiple comparisons was used. For non‐parametric data, the Brown‐Forsythe and Welch ANOVA with Dunnett T3 multiple comparisons was used. For experiments with two variables, Kruskal–Wallis test with Dunn's multiple comparisons between all groups was performed. Test results were considered statistically significant at values of *p* < 0.05 using GraphPad Prism v9.0.

## Conflict of Interest

The authors declare no conflict of interest.

## Supporting information



Supporting Information

## Data Availability

The data that support the findings of this study are available in the supplementary material of this article.
