# Supplementary material for: Precision mRNA Delivery via Ultrasound‐Controlled Release Perfluorocarbon Emulsions: An Innovative Ultrasound Theranostic Strategy with 19F MRI Feasibility
Source: Small. 2025 Nov 28;22(11):e06806. doi: 10.1002/smll.202506806 (PMC12921544; doi:10.1002/smll.202506806)
Supplement: Supplementary file 1 — Supporting Information [file SMLL-22-e06806-s001.docx]

**SUPPORTING INFORMATION**

**Precision mRNA delivery via ultrasound-controlled release perfluorocarbon emulsions: An innovative ultrasound theranostic strategy with ^19^F MRI feasibility**

Haikun Liu,^1,2,3,†^ Mark Louis P. Vidallon,^1,2,3,4,5,†,^* Yuyang Song,^1,2,3^ Aidan P. G. Walsh,^1,2,3,5,6^ Henry Gordon,^1,2,3^ Shulei Ren,^1,2,3^ Pengkai Shi,^1,2,3^ Bangyan Xu,^1,2,3^ Mitchell J. Moon,^2,3,5,6^ Sylvain Trépout,^7^ Rico F. Tabor,^4^ Alexis I. Bishop,^8^ Ulrich Flögel,^9,10^ Karlheinz Peter,^2,3,5,6,11^ and Xiaowei Wang^1,2,3,5,11^*

^1^ Molecular Imaging and NanoTherapeutics Laboratory, Baker Heart and Diabetes Institute, 75 Commercial Road, Melbourne, VIC, 3004, Australia

^2^ Centre for Cardiometabolic mRNA Therapy, Baker Heart and Diabetes Institute, 75 Commercial Road, Melbourne, VIC, 3004, Australia

^3^ Department of Cardiometabolic Health, University of Melbourne, Parkville, VIC, 3010, Australia

^4^ School of Chemistry, Monash University, Clayton, VIC, 3800, Australia

^5^ Baker Department of Cardiovascular Research, Translation and Implementation La Trobe University, Bundoora, VIC, 3086, Australia

^6^ Atherothrombosis and Vascular Biology Laboratory, Baker Heart and Diabetes Institute, 75 Commercial Road, Melbourne, VIC, 3004, Australia

^7^ Ramaciotti Centre for Cryo-electron Microscopy, Monash University, Clayton, VIC, 3800, Australia

^8^ School of Physics and Astronomy, Monash University, Clayton, VIC, 3800, Australia

^9^ Institute for Molecular Cardiology, Medical Faculty and University Hospital Düsseldorf, Heinrich-Heine-University Düsseldorf, Universitätsstraße 1, 40225 Düsseldorf, Germany

^10^ Cardiovascular Research Institute Düsseldorf (CARID), Medical Faculty and University Hospital Düsseldorf, Heinrich-Heine University Düsseldorf, Universitätsstraße 1, 40225 Düsseldorf, Germany

^11^ School of Translational Medicine, Monash University, Melbourne, VIC, 3004, Australia

^†^Equally contributing first authors

*Correspondence to: Dr. Mark Louis P. Vidallon ([marklouis.vidallon@unimelb.edu.au](mailto:marklouis.vidallon@unimelb.edu.au)); Molecular Imaging and NanoTherapeutics Laboratory, Baker Heart and Diabetes Institute, 75 Commercial Road, Melbourne, VIC, 3004, Australia; Prof. Xiaowei Wang ([xiaowei.wang@unimelb.edu.au](mailto:xiaowei.wang@unimelb.edu.au)); Baker Department of Cardiometabolic Health, University of Melbourne, Parkville, VIC, 3010, Australia

**Table S1**. Lipid compositions, droplet size, and ultrasound contrast-enhancing capabilities of different PFCE ND formulations.

| Formulations | | | Characterisation | | |
| --- | --- | --- | --- | --- | --- |
| Total lipid concentration (mg/mL) | DOPE-to-DC-cholesterol mass ratio | PFCE content (% vol) | Diameters (nm) | Mean grey value (px) | Contrast peak time (mins) |
| 3 | 2:1 | 1 | 141.6 | 0.81 | 8-10 |
| 3 | 2:1 | 5 | 221.9 | 50 | 8-10 |
| 4 | 1:1 | 10 | 187.1 | 8.8 | 6-8 |
| 3 | 2:1 | 20 | 295.8 | 250 | 1-3 |
| 3 | 2:1 | 32 | 352.9 | 195 | 7-10 |
| 4 | 1:1 | 40 | 355.5 | 290 | 7-9 |


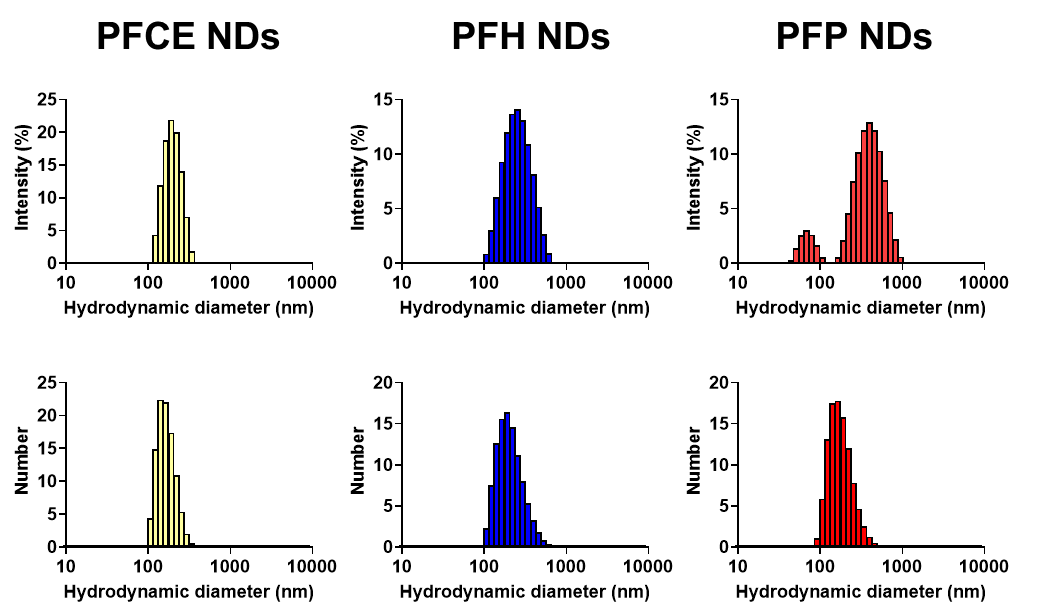


**Figure S1.** DLS size distribution plots of PFC NDs: (*top row*) intensity-weighted distribution and (*bottom row*) number-weighted distribution.


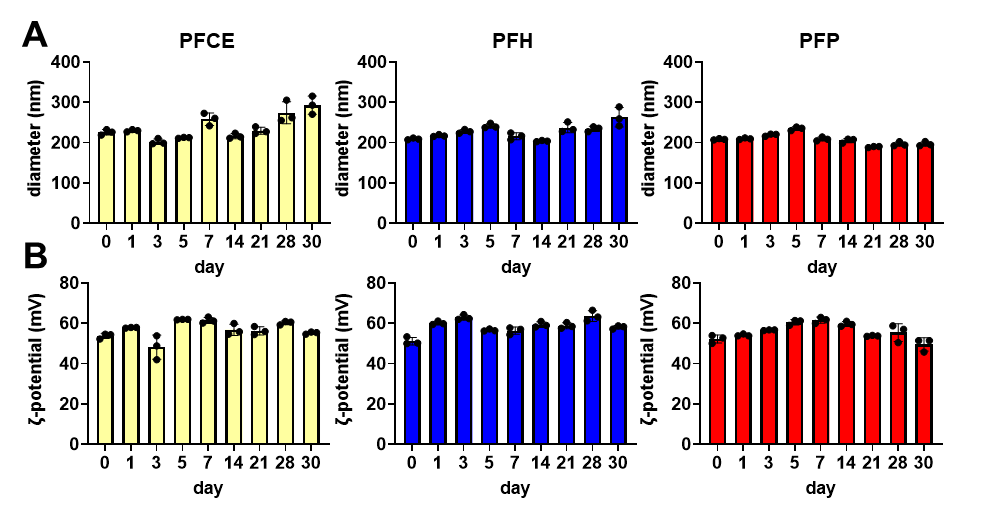


**Figure S2.** Storage stability of PFCE, PFH and PFP NDs for 30 days at 4°C via DLS and ELS monitoring: (**A**) hydrodynamic diameter (particle size) and (**B**) surface charge. Data presented as mean ± SD (*n* = 3); Kruskal-Wallis test with Dunn’s multiple comparisons test, comparing all groups. No significant differences were observed between Day 0 and Day 30.


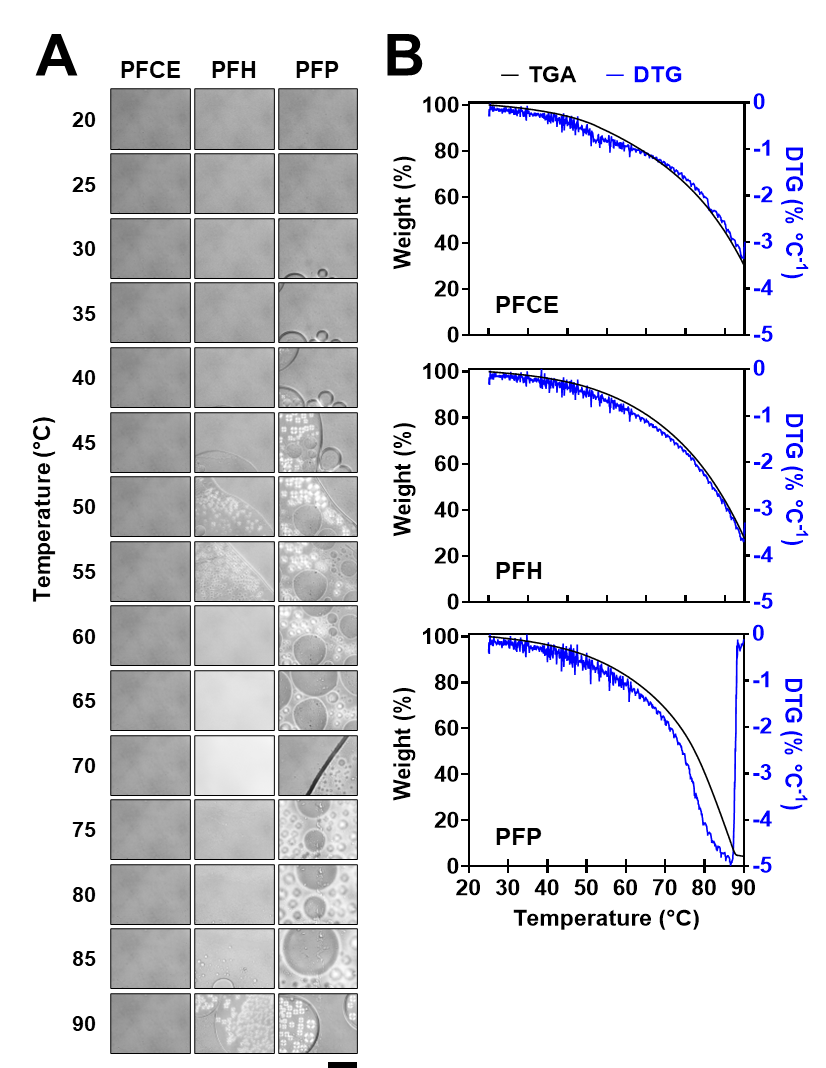


**Figure S3.** (**A**) Representative optical photomicrographs showing the thermal stability and microbubble production from PFC NDs via heating from 20°C to 90°C. Scale bars = 300 µm. (**B**) Thermogravimetric (TGA) and derivative thermogravimetric (DTG) plots of aqueous dispersions of PFC NDs showing differences in thermal stability.


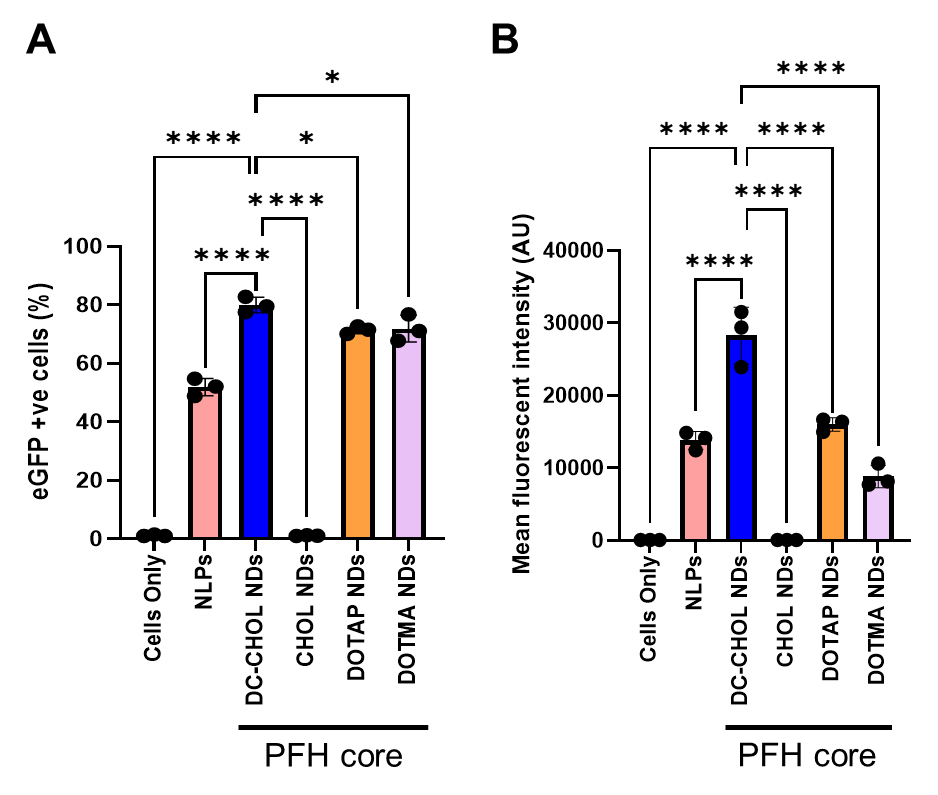
 **Figure S4**. Bar graphs showing the transfection performance of different nanomaterials: PFH NDs with DC-CHOL/DOPE shell, nanoliposomes with DC-CHOL/DOPE shell, PFH NDs with CHOL/DOPE shell, PFH NDs with DOTAP/DOPE shell, and PFH NDs with DOTMA/DOPE shell. (A) Transfection efficiencies in terms of eGFP-positive cells and (B) mean fluorescence intensities, indicating relative protein eGFP expression levels of transfected CHO cells 24-h post-treatment. Bar graphs are shown as mean ± SD from three independent experiments (n = 3), using one-way ANOVA with Tukey’s multiple comparisons; **p* < 0.05, *****p* < 0.0001.


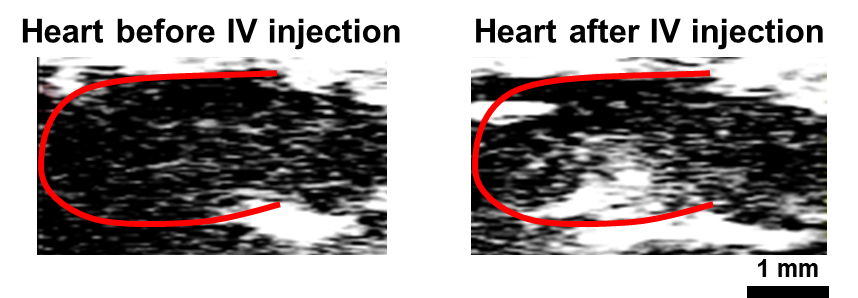


**Figure S5.** B-mode ultrasonograms of mouse chest region (sagittal plane view) showing the atrium region and carotid artery at different stages of treatment: before IV injection, and first observation of NDs just before burst ultrasound application. The red outlines show the atrium region highlighting the appearance of PFH NDs.


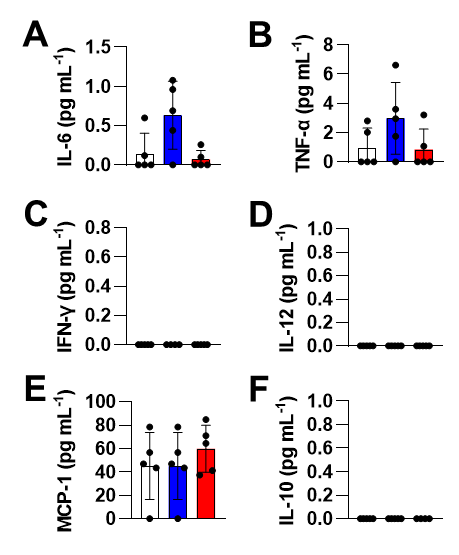


**Figure S6.** Inflammatory cytokine expression in plasma of C57BL/6 mice, collected 24 h post-administration (IV) of mRNA-loaded PFH and PFP NDs: (**A**–**D**) pro-inflammatory, (**E**) chemokine, and (**F**) anti-inflammatory. Data presented as mean ± SD (*n* = 5); Kruskal-Wallis test with Dunn’s multiple comparisons test, comparing all groups; no significant differences were found among groups.
